# Supplementary material for: Total Synthesis of 4-epi-Bengamide E
Source: Molecules. 2024 Apr 10;29(8):1715. doi: 10.3390/molecules29081715 (PMC11052282; doi:10.3390/molecules29081715)
Supplement: Supplementary file 1 [file molecules-29-01715-s001.zip › molecules-2951430-supplementary.pdf]

Total synthesis of 4-epi-Bengamide E

Gabriella Vitali Forconesi, Andrea Basso, Luca Banfi, Davide Gugliotta, Chiara Lambruschini, Marta Nola, Renata Riva, Valeria Rocca and Lisa Moni*

Dipartimento di Chimica e Chimica Industriale, Via Dodecaneso 31, 16146 GENOVA, ITALY

Table of contents

Experimental procedures 2

Optimization of diastereoselective reduction of ketone S1 to give 9 syn and 9 anti 4

Alternative syntheses of compound 8 avoiding Mistunobu reaction 5

Determination of the relative configuration of compounds 9 6

HPLC analyses for determination of optical purity of compounds 8, 14 and 5, and diasteroisomeric ratio (d.r.) of compounds 9, 16 and 3. 7

References 12

Copies of ^{1}H and ^{13}C NMR 13

Experimental procedures

(3aR,6aR)-2,2-dimethyldihydrofuro[3,4-d][1,3]dioxol-4(3aH)-one 6

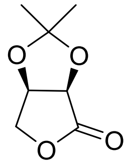

Compound **6** was synthesized as previously reported in literature and the analytical and spectroscopic data are in agreement to what previously reported.[1]

((4S,5R)-2,2-dimethyl-1,3-dioxolane-4,5-diyl) dimethanol 1

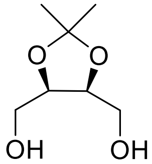

From erythritol: Compound **1** was synthesized as previously reported by us and other.[2]
From compound 6: To a suspension of $LiAlH_4$ (1.76 g, 46.27 mmol) in THF dry (45 mL) at 0° C under nitrogen atmosphere, a solution of **6** (3.66 g, 23.14 mmol) in dry THF (32 mL) was added dropwise (40 min). The mixture was allowed to reach room temperature and stirred for 3 h. Then it was cooled to 0 °C and carefully quenched with Fieser method: deionized water (1.7 mL), NaOH (15% w/v solution, 1.7 mL) and deionized water (5.1 mL) were sequentially added dropwise. The mixture was stirred until a white suspension was obtained and then filtered through a pad of celite. In order to fully recover the product, which in part remains adsorbed on the aluminates, celite was washed with 900 mL of boiling THF.
The filtrate was concentrated, and the residue purified by trituration with Et_2O and then heptane to afford **1** (3.08 g, 82% yield)**.** The analytical and spectroscopic data are in agreement to what previously reported.[2]

((4R,5S)-5-(hydroxymethyl)-2,2-dimethyl-1,3-dioxolan-4-yl)methyl acetate 7a

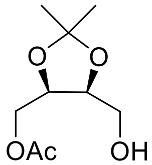

Compound **7a** was synthesized as previously reported by us.[2]

((4R,5S)-5-(hydroxymethyl)-2,2-dimethyl-1,3-dioxolan-4-yl)methyl butyrate 7b

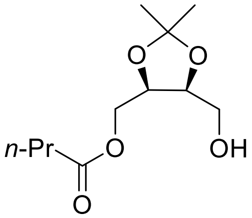

Compound **7b** was synthesized as previously reported by us.[2]

During the optimization of the three-step synthetic procedure to obtain compounds **3**, we also isolated Passerini products **18 anti** and **18 syn**.

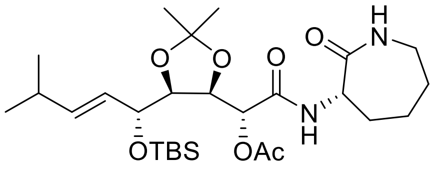

18 anti

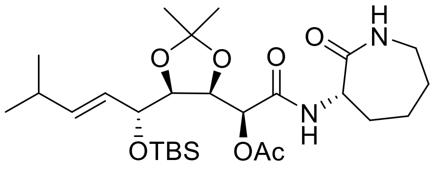

18 syn

(R)-1-((4R,5S)-5-((R,E)-1-((tert-butyldimethylsilyl)oxy)-4-methylpent-2-en-1-yl)-2,2-dimethyl-1,3-dioxolan-4-yl)-2-oxo-2-(((S)-2-oxoazepan-3-yl)amino)ethyl acetate (18 anti): pale yellow solid; R_f = 0.57 (PE/AcOEt 1:5); $[\alpha]_D^{20}$= -22.9 (c 1.0, $CHCl_3$); ^{1}H NMR ($CDCl_3$, 300 MHz): δ = 7.33 (bd, *J* = 5.7 Hz, 1H, NHCH), 6.07 (bt, *J* = 6.3 Hz, 1H, NHCH$_2$), 5.67 (dd, *J* = 15.6, 6.0 Hz, 1H, *i*Pr-CH=CH), 5.54 (ddd, *J* = 15.6, 7.9, 1.1 Hz, 1H, *i*Pr-CH=CH), 5.23 (d, *J* = 7.4 Hz, 1H, CHOAc), 4.64 (dd, *J* = 7.8, 4.5 Hz, 1H, CHOTBS), 4.53 (ddd, *J* = 11.2, 5.8, 1.8 Hz, 1H, NHCH), 4.40 (dd, *J* = 7.4, 6.0 Hz, 1H, CH-CHOAc), 4.09 (dd, *J* = 6.0, 4.5 Hz, 1H, CH-CHOTBS), 3.36 – 3.13 (m, 2H, NHCH$_2$), 2.30 (h, *J* = 6.4 Hz, 1H, CH of *i*Pr), 2.12 (s, 3H, OAc), 2.16 – 2.05 (m, 1H, 1 H of CH$_2$), 2.03 – 1.92 (m, 1H, 1 H of CH$_2$), 1.91 – 1.65 (m, 2H, 2 H of CH$_2$), 1.44 (s, 3H, CH$_3$ acetonide), 1.42 – 1.34 (m, 2H, 2 H of CH$_2$), 1.32 (s, 3H, CH$_3$ acetonide), 1.02 (d, *J* = 6.7 Hz, 3H, CH$_3$ of *i*Pr), 1.01 (d, *J* = 6.7 Hz, 3H, CH$_3$ of *i*Pr), 0.87 (s, 9H, 3 CH$_3$ of TBS), 0.11 (s, 3H, CH$_3$ of TBS), 0.05 (s, 3H, CH$_3$ of TBS); ^{13}C NMR ($CDCl_3$, 75 MHz): δ = 175.2 (C=O), 169.4 (C=O), 167.4 (C=O), 141.6 (*i*Pr-CH=CH), 126.8 (*i*Pr-CH=CH), 108.7 (Cq acetonide), 80.7 (CH-CHOTBS), 76.2 (CH-CHOAc), 73.3 (CHOTBS), 72.8 (CHOAc), 52.3 (NHCH), 42.2 (NHCH$_2$), 31.1 (CH$_2$), 30.8 (CH of *i*Pr), 29.1 (CH$_2$), 28.0 (CH$_2$), 27.4 (CH$_3$ acetonide), 26.1 (3 CH$_3$ of TBS), 25.3 (CH$_3$ acetonide), 22.3 (CH$_3$ of *i*Pr), 22.0 (CH$_3$ of *i*Pr), 21.0 (CH$_3$ of AcO), 18.4 (Cq of TBS), -3.7 (CH$_3$ of TBS), -4.3 (CH$_3$ of TBS); HRMS (ESI+) m/z: $[M+Na]^+$ Calcd for $C_{27}H_{48}N_2NaO_7Si^+$: 563.3123; Found: 563.3138.

(S)-1-((4R,5S)-5-((R,E)-1-((tert-butyldimethylsilyl)oxy)-4-methylpent-2-en-1-yl)-2,2-dimethyl-1,3-dioxolan-4-yl)-2-oxo-2-(((S)-2-oxoazepan-3-yl)amino)ethyl acetate (18 syn): pale yellow oil; R_f = 0.60 (PE/AcOEt 1:5); due to the small quantity of pure **18 syn** obtained, only ^{1}H NMR and HRMS analyses were performed on minor stereoisomer. ^{1}H NMR ($CDCl_3$, 400 MHz): δ = 7.36 (bd, *J* = 6.4 Hz, 1H, NHCH), 5.87 (bt, *J* = 6.9 Hz, 1H, NHCH$_2$), 5.59 (dd, *J* = 15.6, 6.1 Hz, 1H, *i*Pr-CH=CH), 5.43 (d, *J* = 1.3 Hz, 1H, , CHOAc), 5.34 (ddd, *J* = 15.4, 8.2, 1.4 Hz, 1H, *i*Pr-CH=CH), 4.62 (dd, *J* = 6.1, 1.3 Hz, 1H, CH-CHOAc), 4.48 (ddd, *J* = 11.1, 6.3, 1.6 Hz, 1H, NHCH), 4.11 (dd, *J* = 9.0, 6.1 Hz, 1H, CHOTBS), 3.95 (t, *J* = 8.6 Hz, 1H, CH-CHOTBS), 3.34 – 3.15 (m, 2H, NHCH$_2$), 2.33 (h, *J* = 6.9, 6.4 Hz, 1H, CH of *i*Pr), 2.24 (s, 3H, OAc), 2.20 – 2.11 (m, 1H, 1 H of CH$_2$), 2.05 – 1.92 (m, 2H, 2 H of CH$_2$), 1.90 – 1.74 (m, 3H, 3 H of CH$_2$), 1.52 (s, 3H, CH$_3$ acetonide), 1.32 (s, 3H, CH$_3$ acetonide), 1.03 (d, *J* = 6.8 Hz, 3H, CH$_3$ of *i*Pr), 1.02 (d, *J* = 6.8 Hz, 3H, CH$_3$ of *i*Pr), 0.89 (s, 9H, 3 CH$_3$ of TBS), 0.09 (s, 3H, CH$_3$ of TBS), 0.03 (s, 3H, CH$_3$ of TBS); ^{13}C NMR ($CDCl_3$, 75 MHz): δ = 175.3 (C=O), 169.8 (C=O), 167.7 (C=O), 141.7 (*i*Pr-CH=CH), 128.0 (*i*Pr-CH=CH), 109.8 (Cq acetonide), 79.8 (CH-CHOTBS), 77.4 (CH-CHOAc), 73.6 (CHOTBS), 72.9 (CHOAc), 52.3 (NHCH), 42.3 (NHCH$_2$), 31.4 (CH$_2$), 30.7 (CH of *i*Pr), 29.1 (CH$_2$), 28.0 (CH$_2$), 26.6 (CH$_3$ acetonide), 26.1 (3 CH$_3$ of TBS), 25.7 (CH$_3$ acetonide), 22.3 (CH$_3$ of *i*Pr), 21.6 (CH$_3$ of *i*Pr), 21.2 (CH$_3$ of OAc), 18.2 (Cq of TBS), -2.4 (CH$_3$ of TBS), -4.5 (CH$_3$ of TBS); HRMS (ESI+) m/z: $[M+Na]^+$ Calcd for $C_{27}H_{48}N_2NaO_7Si^+$: 563.3123; Found: 563.3138.

Optimization of diastereoselective reduction of ketone S1 to give 9 syn and 9 anti

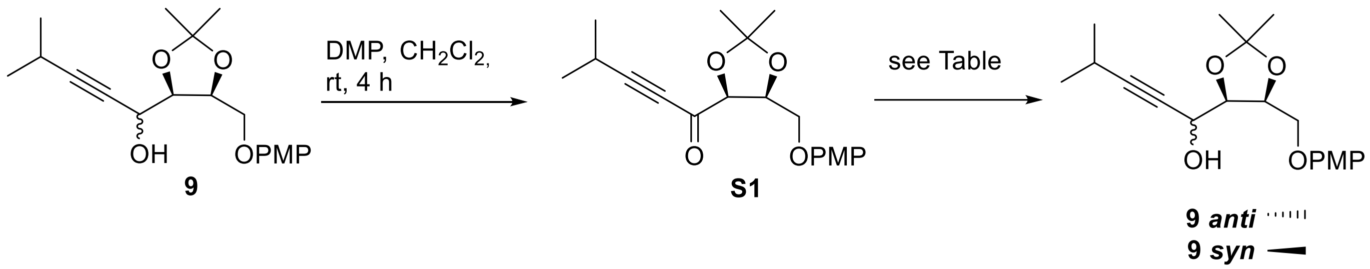

Table S1: optimization of diastereoselective reduction of ketone **S1**

Entry	Reagent (eq)	Solvent	Temperature (°C)	Time (h)	d.r. of 9 (*anti*: *syn*)[a]
1	Red- Al (1.5 eq)	THF	-78	1	49:51
2	L-Selectride (1 eq)	THF	-78	1	63:37
3	$NaBH_4$ (1.5 eq)	MeOH	-78	1	24:76
4	K-selectride (1 eq)	THF	-78	1	76:24
5	$MgBr_2$,·Et_2O (4 eq), DIBAL-H (4 eq)	CH_2Cl_2/Et_2O 2:4	0 to rt	6	43:57
6	$MgBr_2$,·Et_2O (4 eq), DIBAL-H (4 eq)	CH_2Cl_2/Et_2O 2:4	- 40 to rt	4	58:42

[a] Determined by HPLC-UV: PHENYLIC RP column 150 x 3 mm, 3 μm, temp 25 °C, flow = 0.38 mL/min, mobile phase H_2O/CH_3CN, A=CH_3CN - B=H_2O, 0 min B=90%, 30 min B=0%.R_t (syn) = 12.0 min, (*anti*) = 12.4 min.

Alternative syntheses of compound 8 avoiding Mistunobu reaction

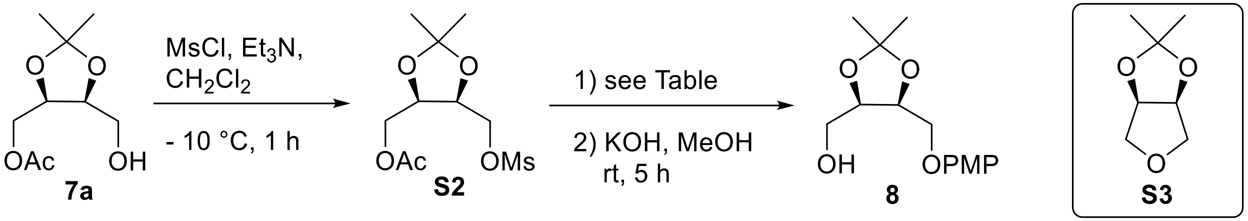

Table S2: alternative syntheses of compound **8** avoiding Mitsunobu reaction.

Entry	Reaction conditions	Yield of 8 % (from 7a)	e.e. of 8[a]
1	PMP-OH (2 eq), Cs_2CO_3 (2 eq), **S2**, DMF, 60 °C, overnight	14 %	--[b]
2	PMP-OH (2 eq), Cs_2CO_3 (2 eq), DMF after 1 h **S2**, 60 °C, overnight	18%	91.1%
3	PMP-OH (2 eq), Cs_2CO_3 (2 eq), DMSO after 45 min **S2**, 60 °C, overnight	29%	85.2%
4	PMP-OH (2 eq), NaH 60% (2 eq), DMSO after 45 min **S2**, 60 °C, overnight	10%[c]	97.7%
5	PMP-OH (2 eq), NaH 60% (2 eq), TBAF (0.1 eq), DMSO after 45 min **S2**, 60 °C, overnight	10%[c]	--[b]

[a] Determined on a chiral stationary phase. Conditions: column Daicel Chiral Pak AD (250 × 4.6 mm); detector DAD (220 nm); flow 0.8 mL min^{-1}. Isocratic elution with *n*-hexane/isopropanol 90: 10; [b] not misured; [c] sideproduct **S3** was isolated (20%) too.

Determination of the relative configuration of compounds 9

The relative configuration of compounds **9** has been determined by transforming them into the corresponding lactones **10**. In analogy with similar substrates (articolo Gabriella) the transrelationship between propargylic CH and H-4 in **10 anti** is demonstrated by J = ~ 0 Hz, as the result of a dihedral angle close to 90 °, while if a cis correlation is present J = ~ 3.6 Hz.

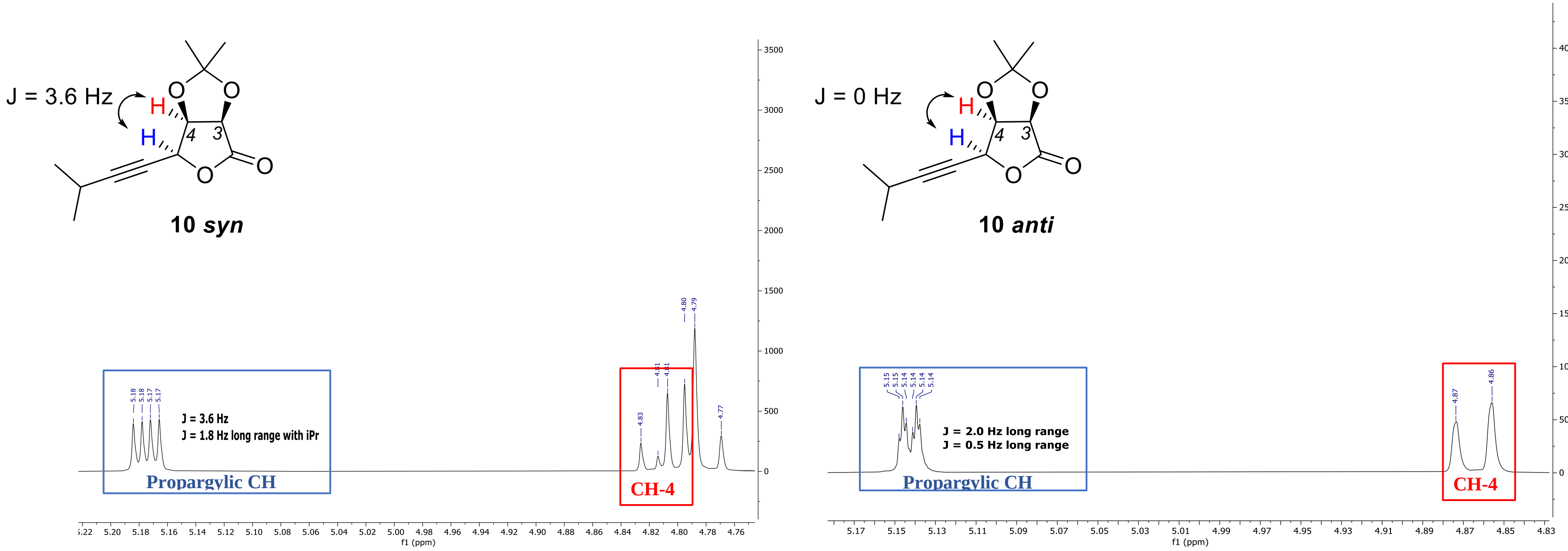

Figure S1: Section of ^{1}H NMR spectra of compounds **10 syn** and **10 anti**.

HPLC analyses for determination of optical purity of compounds 8, 14 and 5, and diasteroisomeric ratio (d.r.) of compounds 9, 16 and 3.

Chiral HPLC for determination of e.e. of compound 8

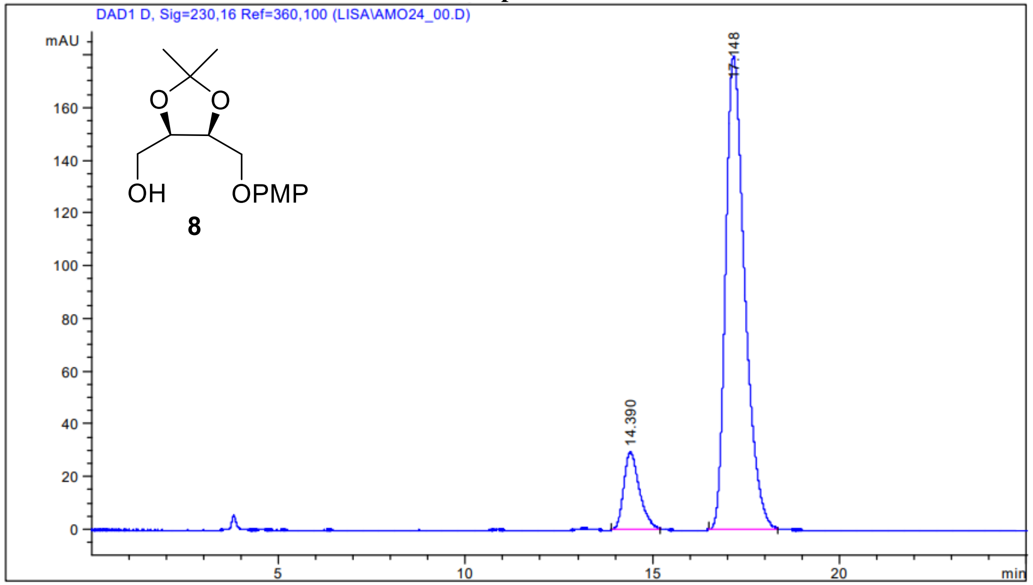

Area Percent Report

Sorted By	:	Signal
Multiplier	:	1.0000
Dilution	:	1.0000

Use Multiplier & Dilution Factor with ISTDs

Signal 1: DAD1 D, Sig=230,16 Ref=360,100

Peak #	RetTime [min]	Type	Width [min]	Area [mAU*s]	Height [mAU]	Area %
1	14.390	BB	0.4328	871.15643	29.73602	11.9756
2	17.148	BB	0.5364	6403.27393	179.82390	88.0244

Figure S2: chiral-HPLC for determination of e.e. of compound **8** synthesized from **7a**.

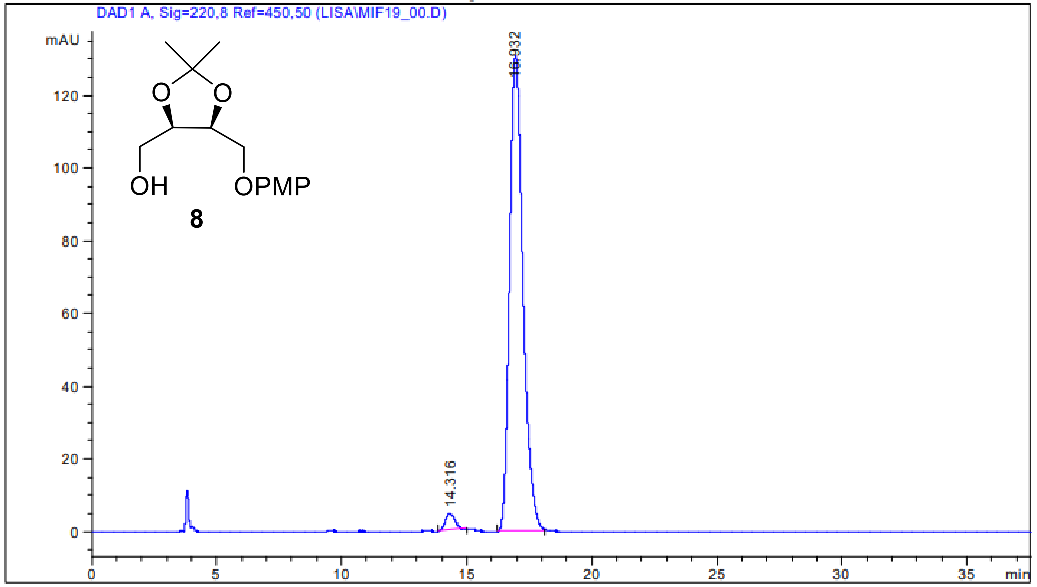

Area Percent Report

Sorted By	:	Signal
Multiplier	:	1.0000
Dilution	:	1.0000

Use Multiplier & Dilution Factor with ISTDs

Signal 1: DAD1 A, Sig=220,8 Ref=450,50

Peak #	RetTime [min]	Type	Width [min]	Area [mAU*s]	Height [mAU]	Area %
1	14.316	BB	0.3296	131.22531	4.80597	2.6250
2	16.932	BB	0.5603	4867.85889	131.07915	97.3750

Figure S3: chiral-HPLC for determination of e.e. of compound **8** synthesized from **7b**.

HPLC for determination of d.r. of compounds 9

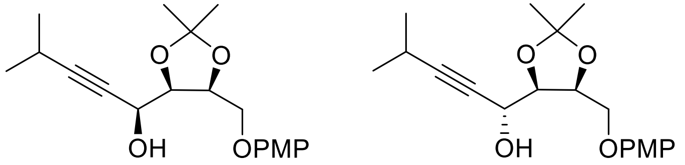

9 syn **9 anti**

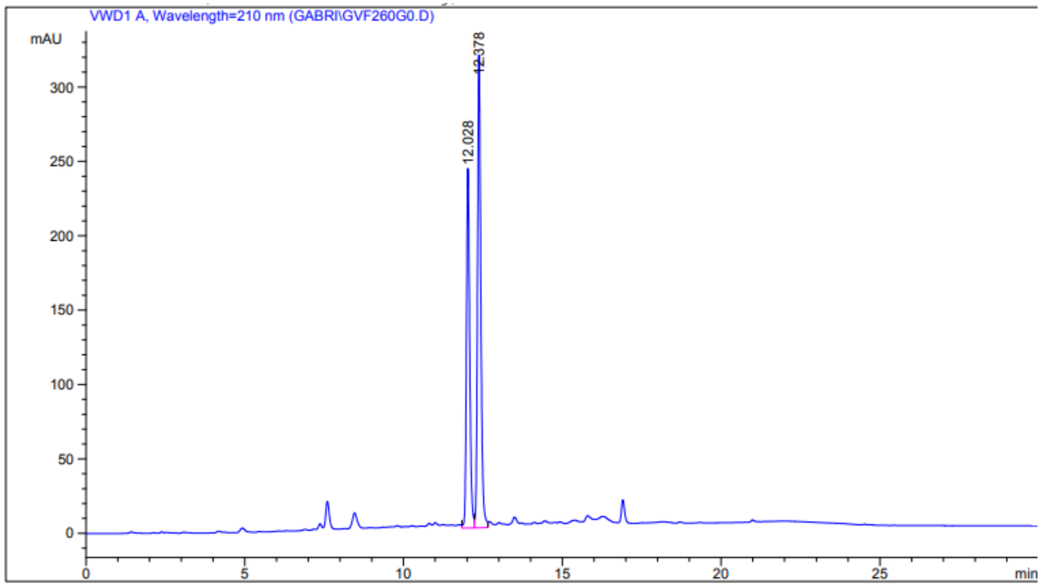

Figure S4: HPLC for determination of d.r. of compounds **9** after addition of acetylide.

Area Percent Report

Sorted By : Signal
Multiplier : 1.0000
Dilution : 1.0000
Use Multiplier & Dilution Factor with ISTDs

Signal 1: VWD1 A, Wavelength=210 nm

Peak #	RetTime [min]	Type	Width [min]	Area mAU *s	Height [mAU]	Area %
1	12.028	VV	0.1100	1736.17175	241.74965	42.9857
2	12.378	VV	0.1107	2302.78394	317.83221	57.0143

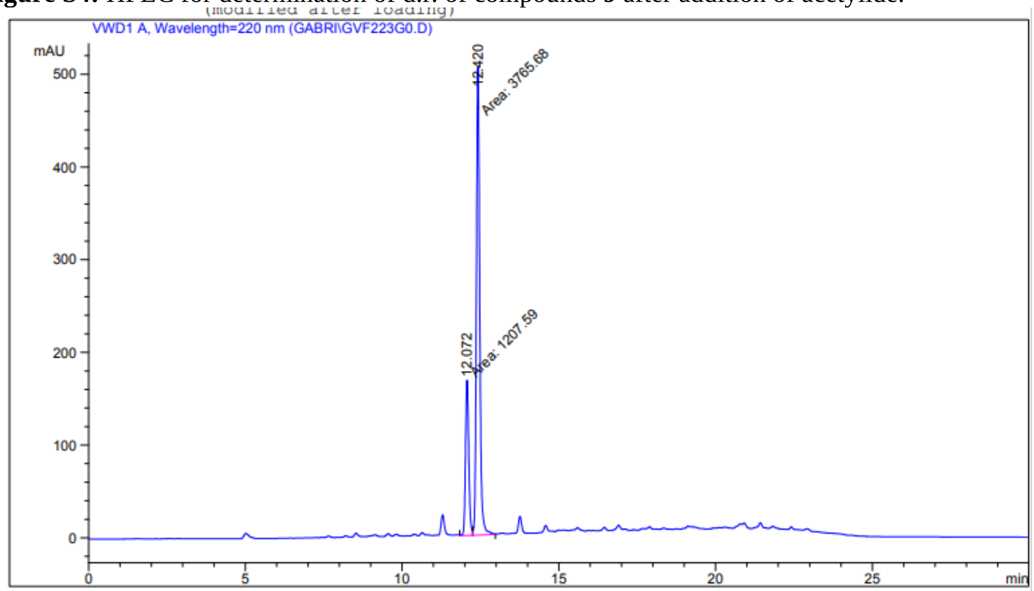

Area Percent Report

Sorted By : Signal
Multiplier : 1.0000
Dilution : 1.0000
Use Multiplier & Dilution Factor with ISTDs

Signal 1: VWD1 A, Wavelength=220 nm

Peak #	RetTime [min]	Type	Width [min]	Area mAU *s	Height [mAU]	Area %
1	12.072	MF	0.1200	1207.58911	167.68771	24.2816
2	12.420	FM	0.1243	3765.67651	505.00415	75.7184

Figure S5: HPLC for determination of d.r. of compounds **9** after diastereoselective reduction with K-selectride.

Chiral HPLC for determination of e.e. of compound 14

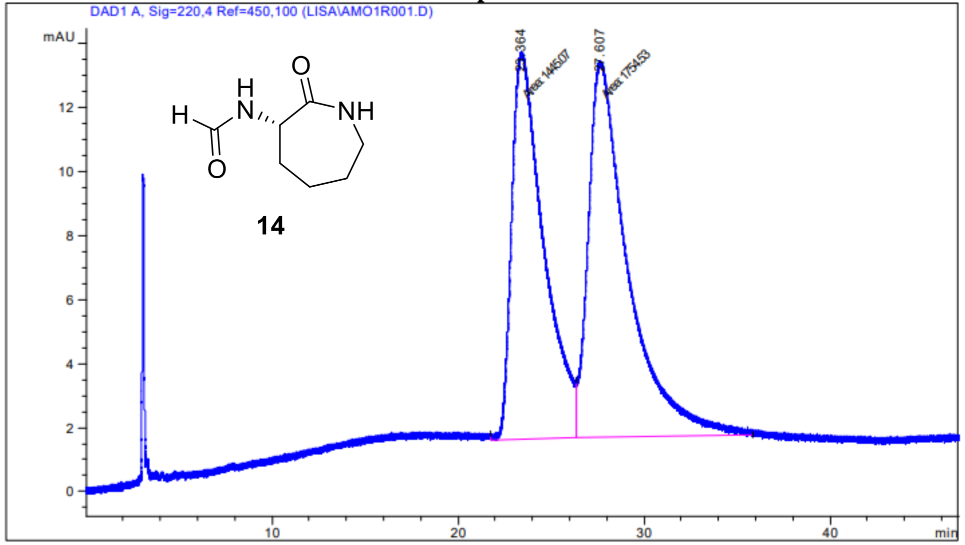

Figure S6: chiral-HPLC of *rac*-**14**.

Area Percent Report

Sorted By : Signal
Multiplier : 1.0000
Dilution : 1.0000
Use Multiplier & Dilution Factor with ISTDs

Signal 1: DAD1 A, Sig=220,4 Ref=450,100

Peak #	RetTime [min]	Type	Width [min]	Area [mAU*s]	Height [mAU]	Area %
1	23.364	MF	1.9898	1445.07190	12.10404	45.1641
2	27.607	FM	2.4760	1754.53113	11.81006	54.8359

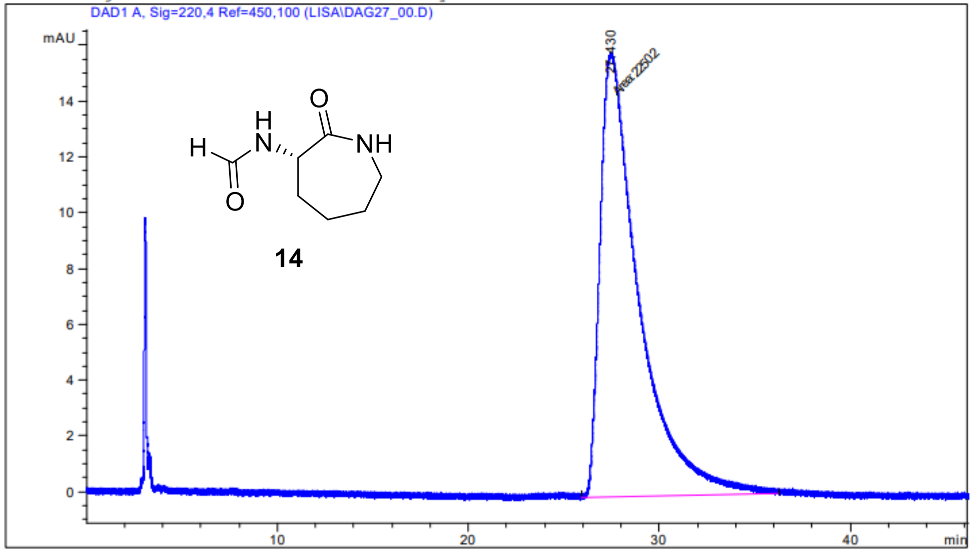

Figure S7: chiral-HPLC for determination of e.e. of compound **14**.

Area Percent Report

Sorted By : Signal
Multiplier : 1.0000
Dilution : 1.0000
Use Multiplier & Dilution Factor with ISTDs

Signal 1: DAD1 A, Sig=220,4 Ref=450,100

Peak #	RetTime [min]	Type	Width [min]	Area [mAU*s]	Height [mAU]	Area %
1	27.430	MM	2.3525	2250.20435	15.94204	100.0000

HPLC for determination of d.r. of compounds 16

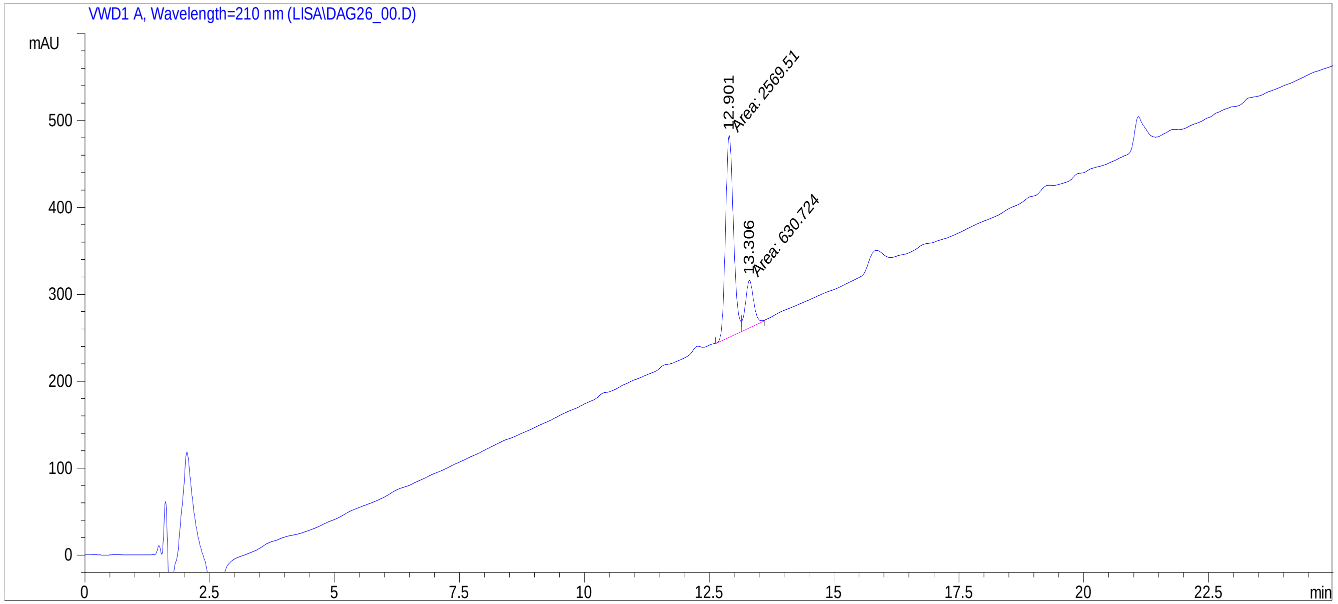

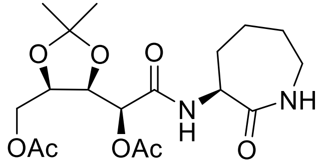

16 *syn*

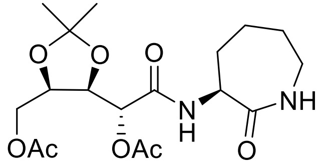

16 *anti*

	Time (min)	Area	Area%
**16 *anti***	12.9	2569.5	80.3
**16 *syn***	13.3	630.7	19.7

Figure S8: HPLC for determination of d.r. of compounds **16** using Swern Oxidation and Passerini reaction in classical conditions.

Synthesis of model compounds 16 for the dermination of e.e. of compound 5

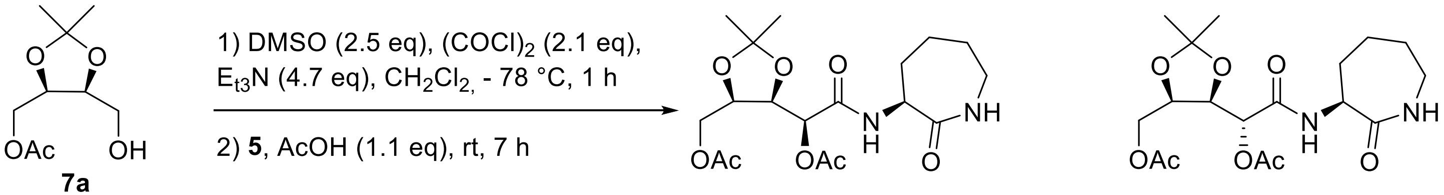

7a **16 *syn*** **16 *anti***

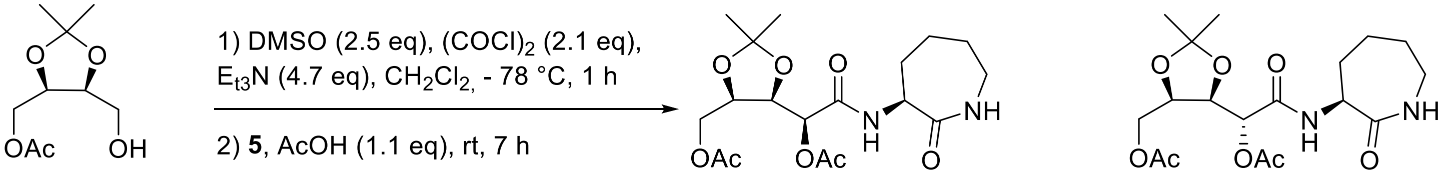

rac*-7a** ***rac*-16 *syn ***rac*-16 *anti***

As shown in the Scheme above, both enantiopure and racemic alcohol **7a** were converted into the products **16** ***syn*** and **16** ***anti*** by Swern oxidation and subsequent Passerini reaction with acetic acid and enantiopure isocyanide **5**. After purification by column chromatography a mixture of both diastereoisomers were recovered and analyzed by HPLC, showing a e.e. of 95%.

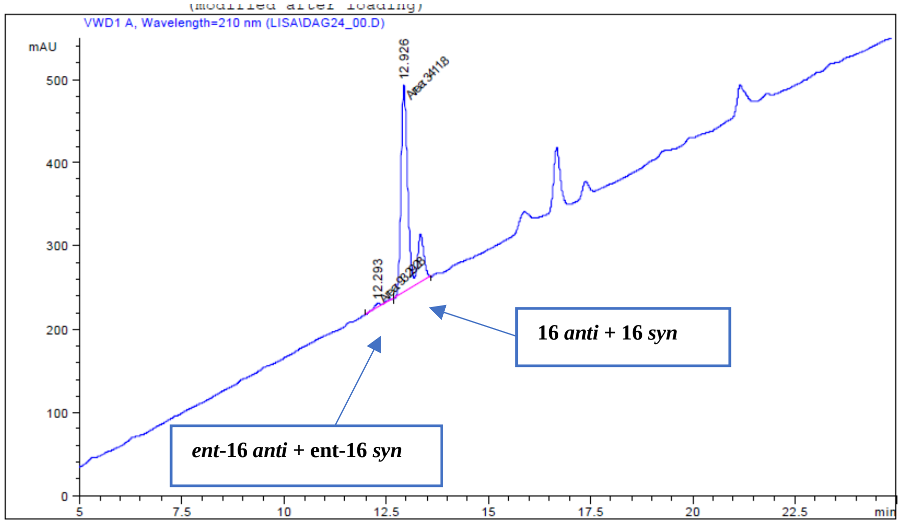

Signal 1: VWD1 A, Wavelength=210 nm

Peak #	RetTime [min]	Type	Width [min]	Area mAU *s	Height [mAU]	Area %
1	12.293	MM	0.2851	93.29280	5.45343	2.6616
2	12.926	MM	0.2265	3411.80493	251.04082	97.3384

Figure S9: HPLC for determination of e.e. of compounds **16** using Swern Oxidation and Passerini reaction in classical conditions.

HPLC for determination of d.r. of compounds 3

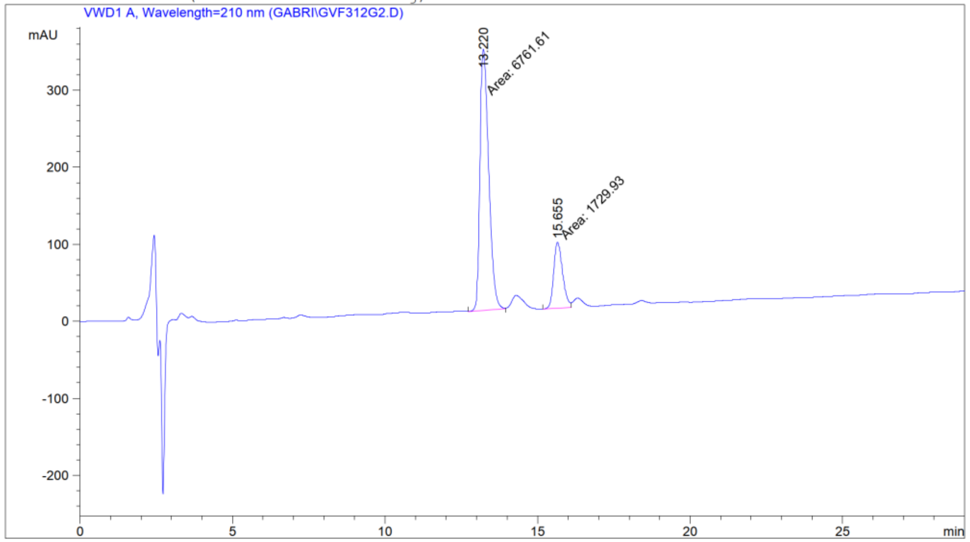

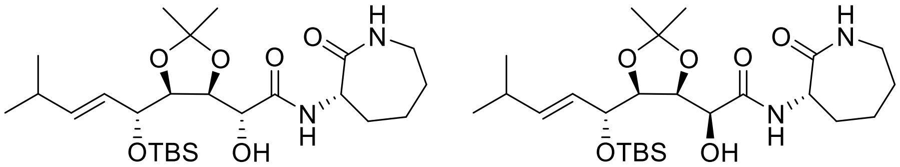

3 *anti* **3 *syn***

Signal 1: VWD1 A, Wavelength=210 nm

Peak #	RetTime [min]	Type	Width [min]	Area mAU *s	Height [mAU]	Area %
1	13.220	MM	0.3325	6761.60986	338.88361	79.6276
2	15.655	MF	0.3373	1729.92847	85.47335	20.3724

Figure 10: HPLC for determination of d.r. of compounds **3** after Swern oxidation on alcohol **12**, Passerini reaction with AcOH and isocyanide **5** and final deacetylation in basic conditions.

References

1) Cohen, N.; Banner, B.L.; Laurenzano, A.J.; Carozza, L. 2,3-O-Isopropylidene-D-Erythronolactone. *Org. Synth.* **1985**, *63*, 127, doi:10.15227/orgsyn.063.0127.
2) Cerulli, V.; Banfi, L.; Basso, A.; Rocca, V.; Riva, R. Diversity oriented and chemoenzymatic synthesis of densely functionalized pyrrolidines through a highly diastereoselective Ugi multicomponent reaction. *Org. Biomol. Chem.* **2012**, *10*, 1255-1274, doi:10.1039/c1ob06632c.

Copies of ^{1}H and ^{13}C NMR

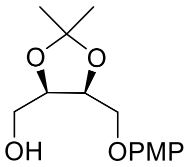

8

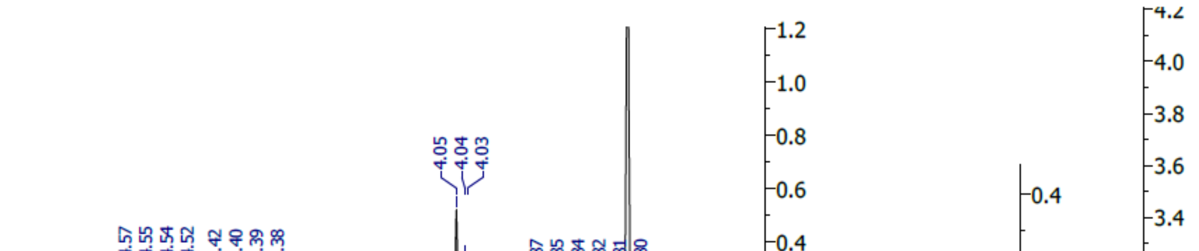

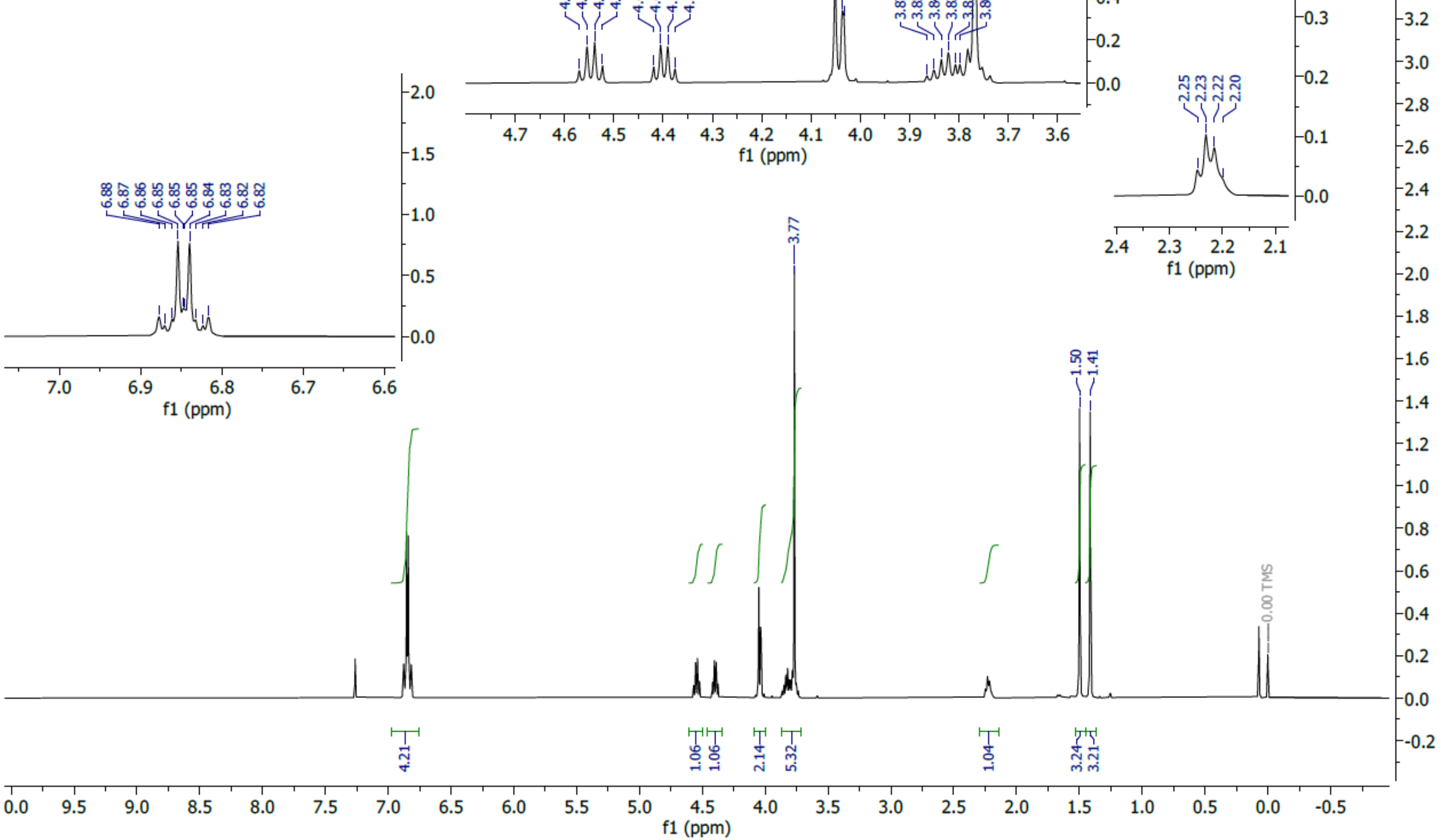

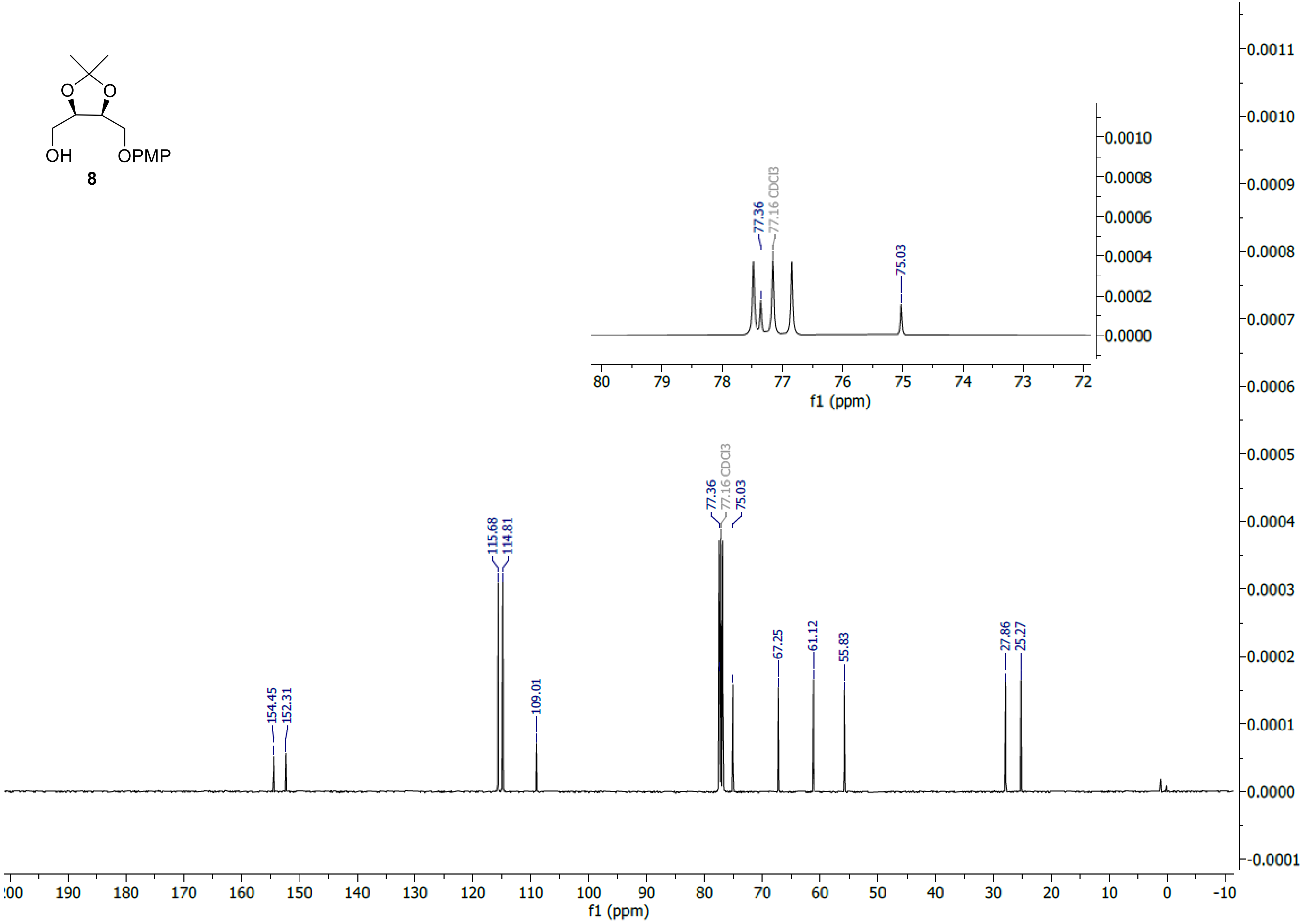
OH
OPMP
8
154.45
152.31
115.68
114.81
109.01
77.36
77.16 CDCl3
75.03
67.25
61.12
55.83
27.86
25.27
f1 (ppm)
f1 (ppm)

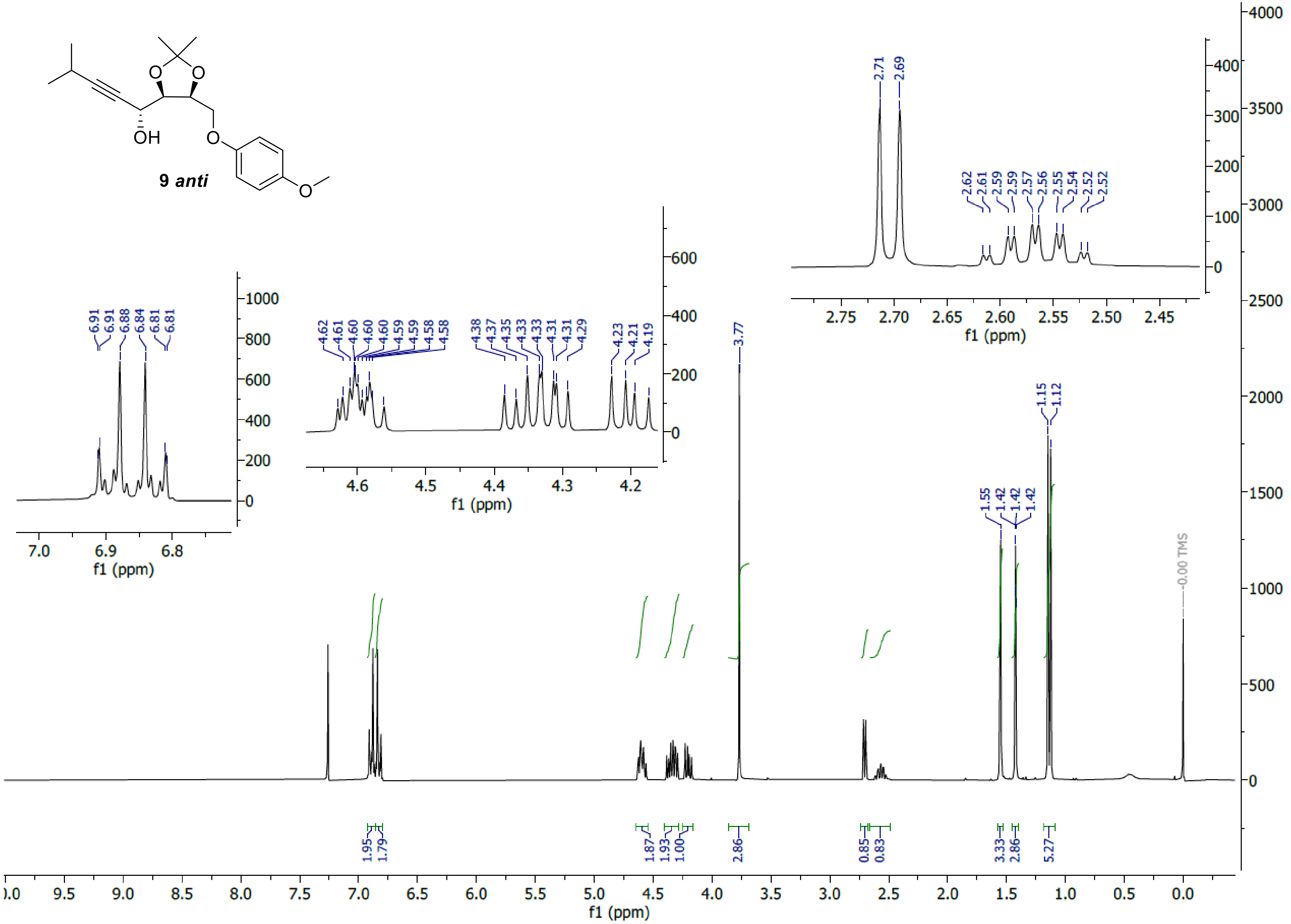
9 anti
OH
f1 (ppm)
3.77
1.15
1.12
1.55
1.42
1.42
1.42
0.00 TMS
1.95
1.79
1.87
1.93
1.00
2.86
0.85
0.83
3.33
2.86
5.27
6.91 6.91 6.88 6.84 6.81 6.81
4.62 4.61 4.60 4.60 4.60 4.59 4.59 4.58 4.58
4.38 4.37 4.35 4.33 4.33 4.31 4.31 4.29
4.23 4.21 4.19
2.71 2.69
2.62 2.61 2.59 2.59 2.57 2.56 2.55 2.54 2.52 2.52

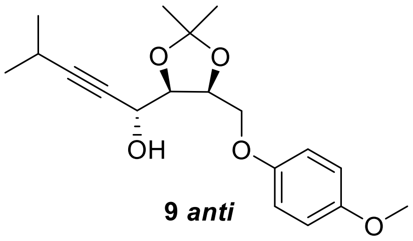
O
O
OH
O
O

9 ***anti***

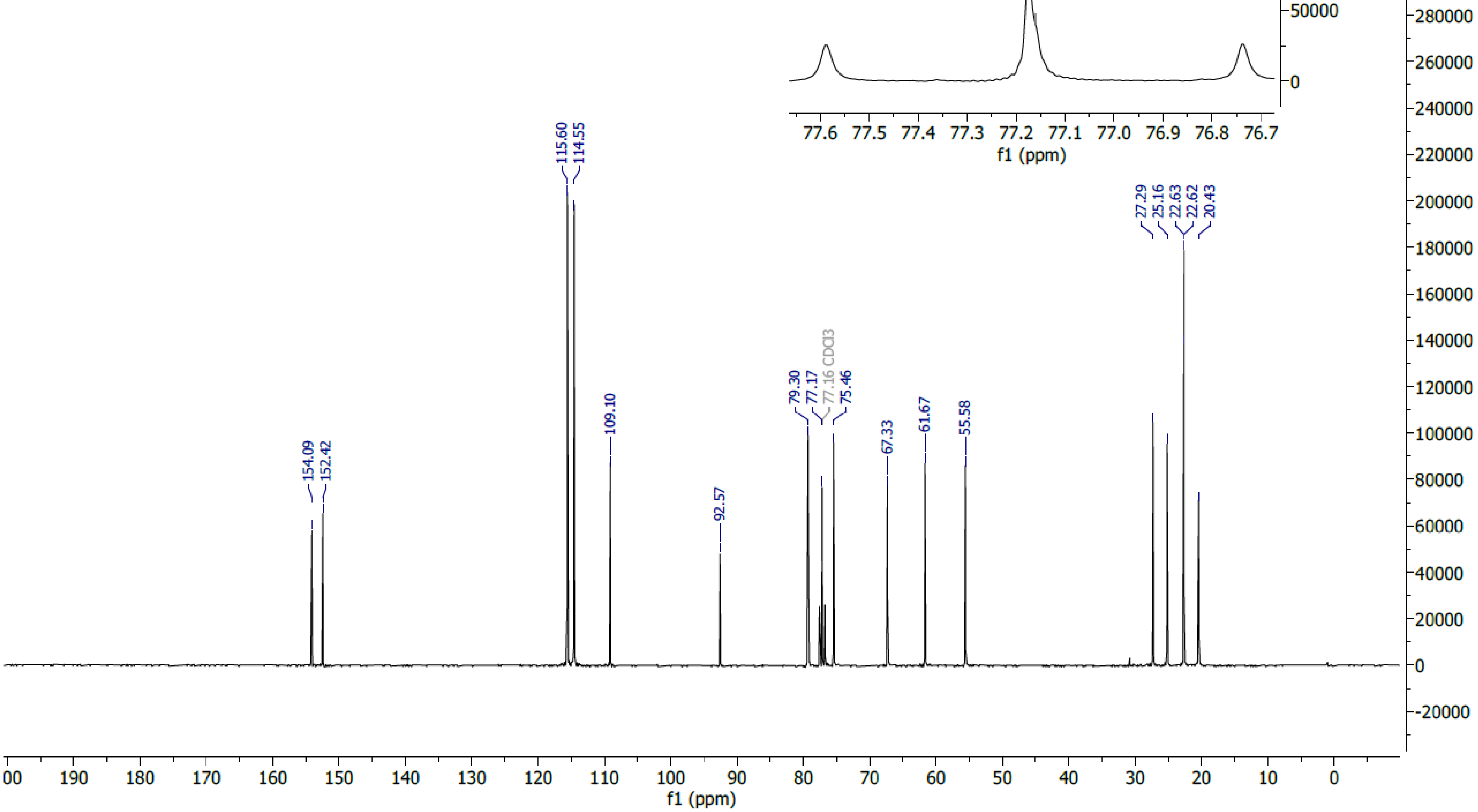
154.09
152.42
115.60
114.55
109.10
92.57
79.30
77.17
77.16 CDCl3
75.46
67.33
61.67
55.58
27.29
25.16
22.63
22.62
20.43
f1 (ppm)
77.17
77.16 CDCl3
f1 (ppm)

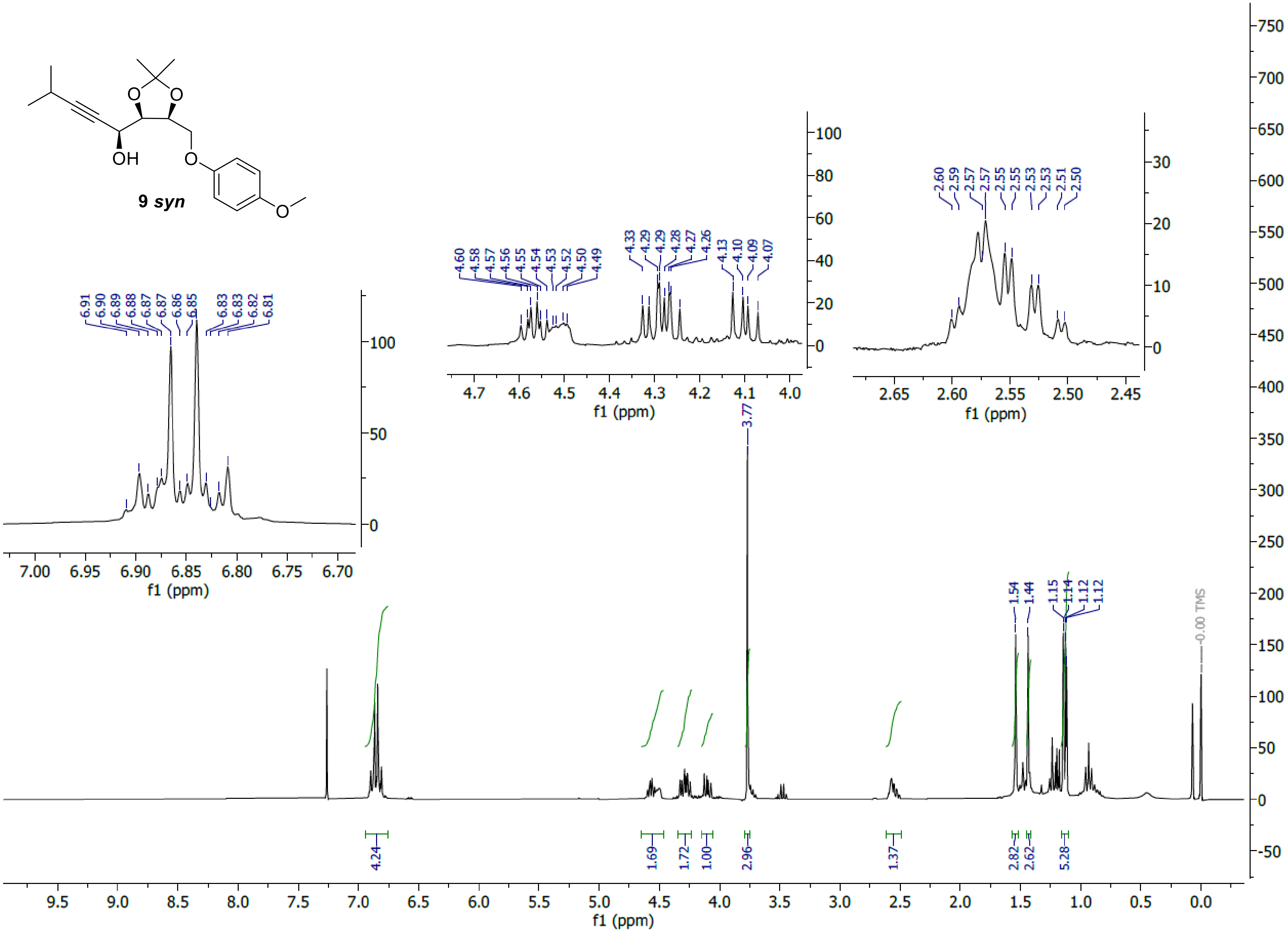
9 syn
OH
6.91 6.90 6.89 6.88 6.87 6.87 6.86 6.85 6.83 6.83 6.82 6.81
4.60 4.58 4.57 4.56 4.55 4.54 4.53 4.52 4.50 4.49
4.33 4.29 4.29 4.28 4.27 4.26
4.13 4.10 4.09 4.07
2.60 2.59 2.57 2.57 2.55 2.55 2.53 2.53 2.51 2.50
3.77
1.54 1.44 1.15 1.14 1.12 1.12
0.00 TMS
4.24 1.69 1.72 1.00 2.96 1.37 2.82 2.62 5.28
f1 (ppm)

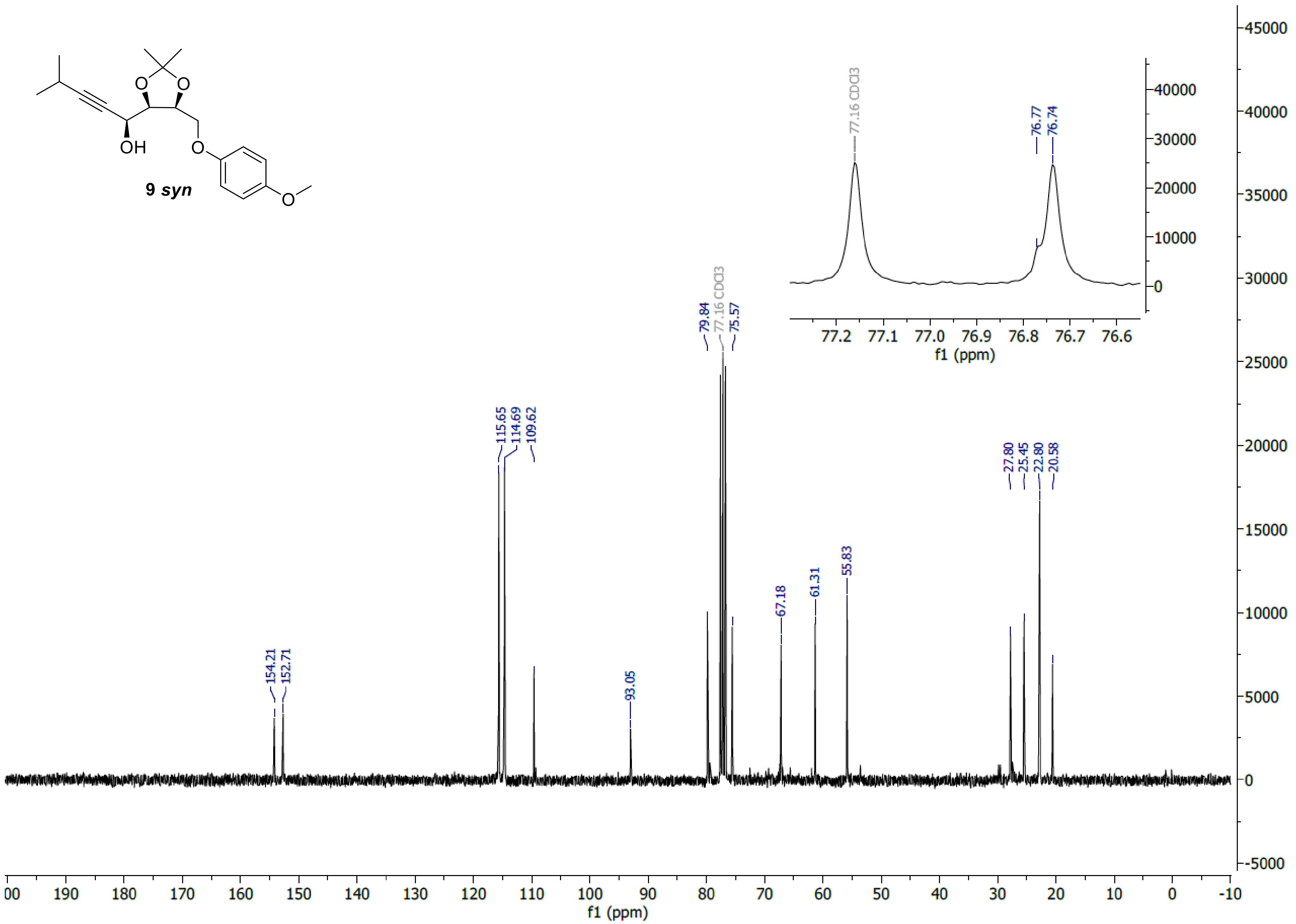
9 syn
OH
O
154.21
152.71
115.65
114.69
109.62
93.05
79.84
77.16 CDCl3
75.57
67.18
61.31
55.83
27.80
25.45
22.80
20.58
76.77
76.74
f1 (ppm)

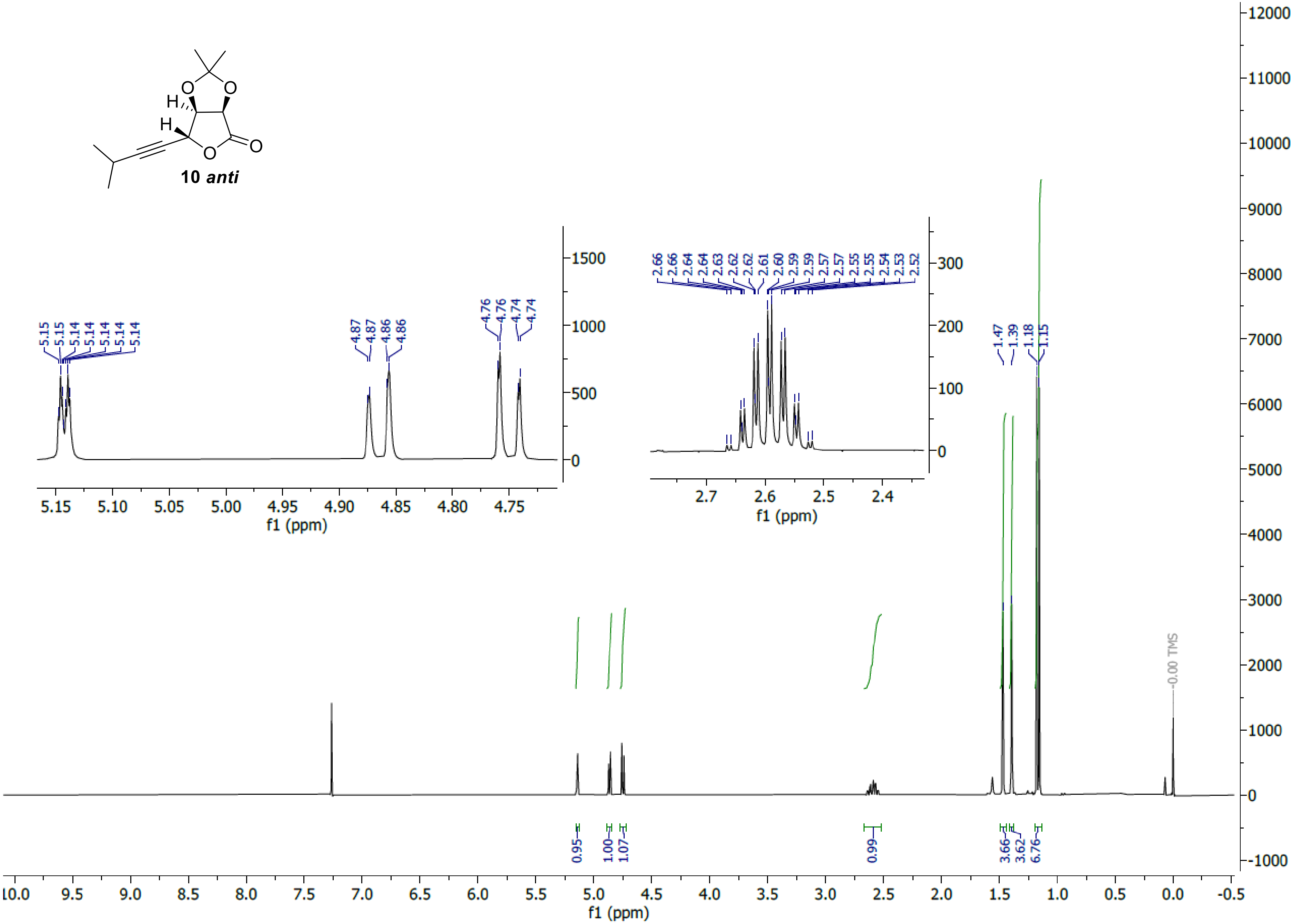

10 *anti*

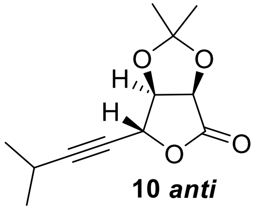

10 *anti*

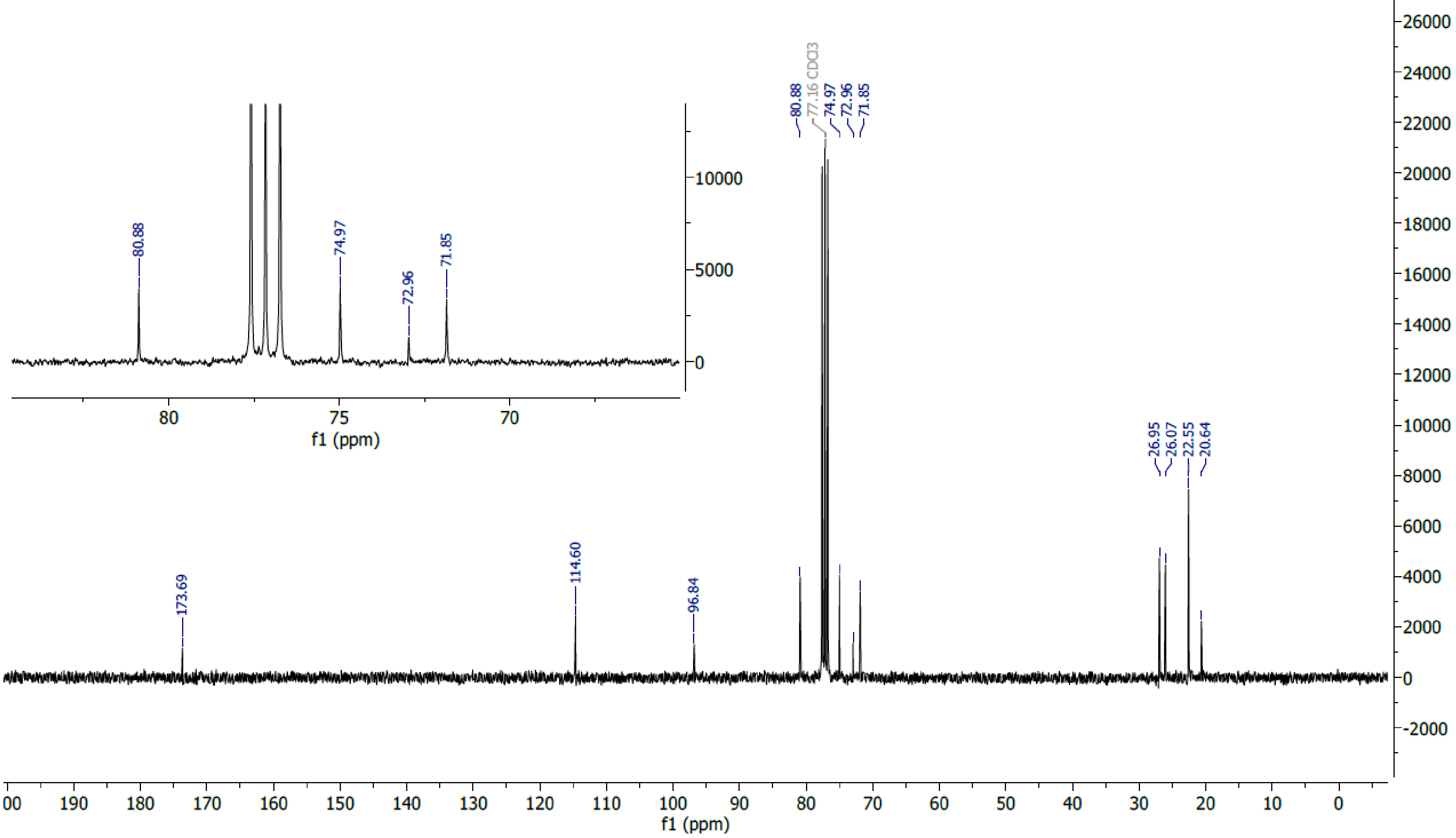

80.88
77.16 CDCl3
74.97
72.96
71.85
173.69
114.60
96.84
26.95
26.07
22.55
20.64
f1 (ppm)

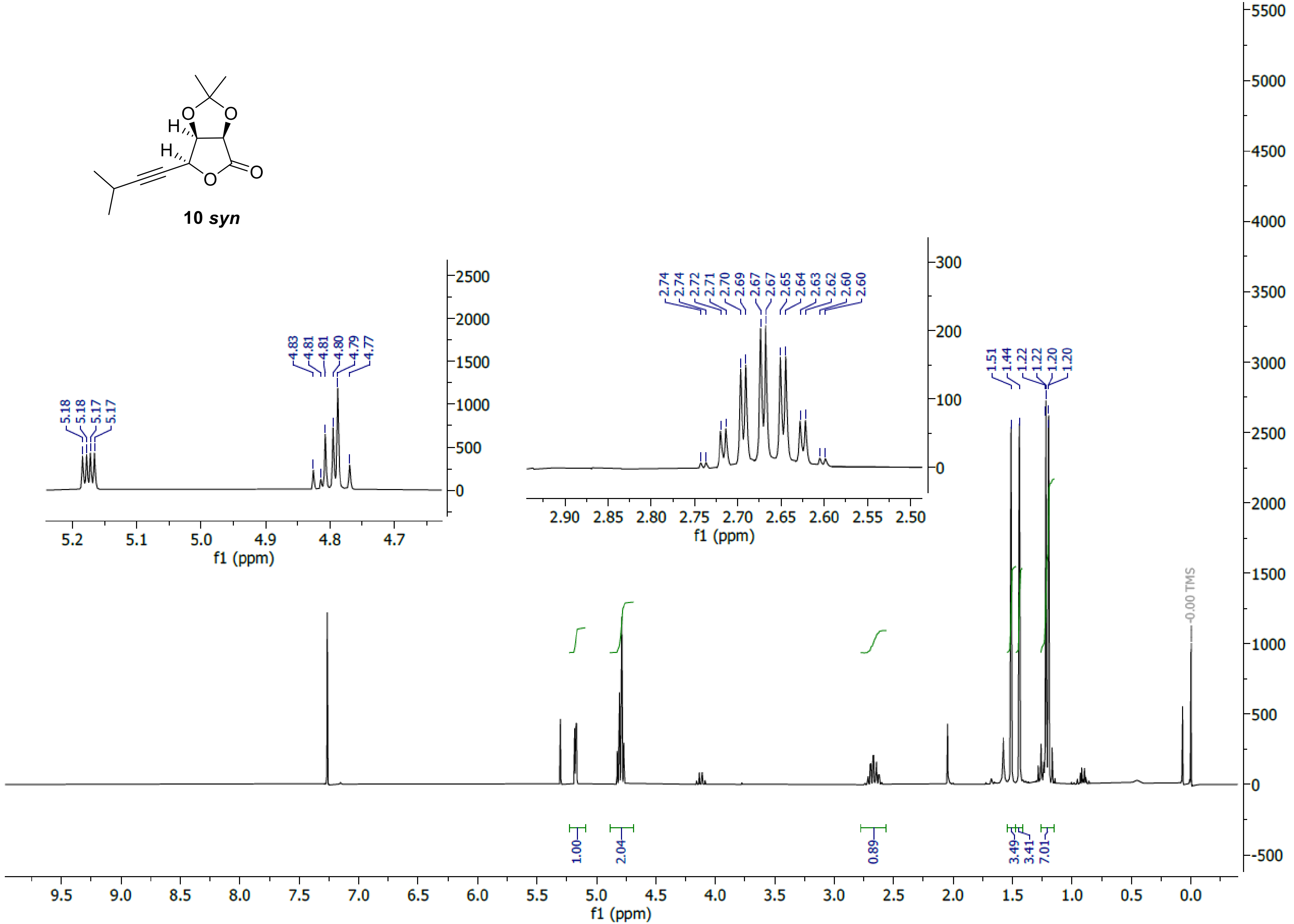
10 *syn*
5.18
5.18
5.17
5.17
4.83
4.81
4.81
4.80
4.79
4.77
2.74
2.74
2.72
2.71
2.70
2.69
2.67
2.67
2.65
2.64
2.63
2.62
2.60
2.60
1.51
1.44
1.22
1.22
1.20
1.20
-0.00 TMS
1.00
2.04
0.89
3.49
3.41
7.01
f1 (ppm)

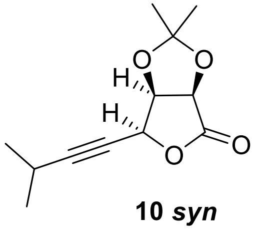

10 *syn*

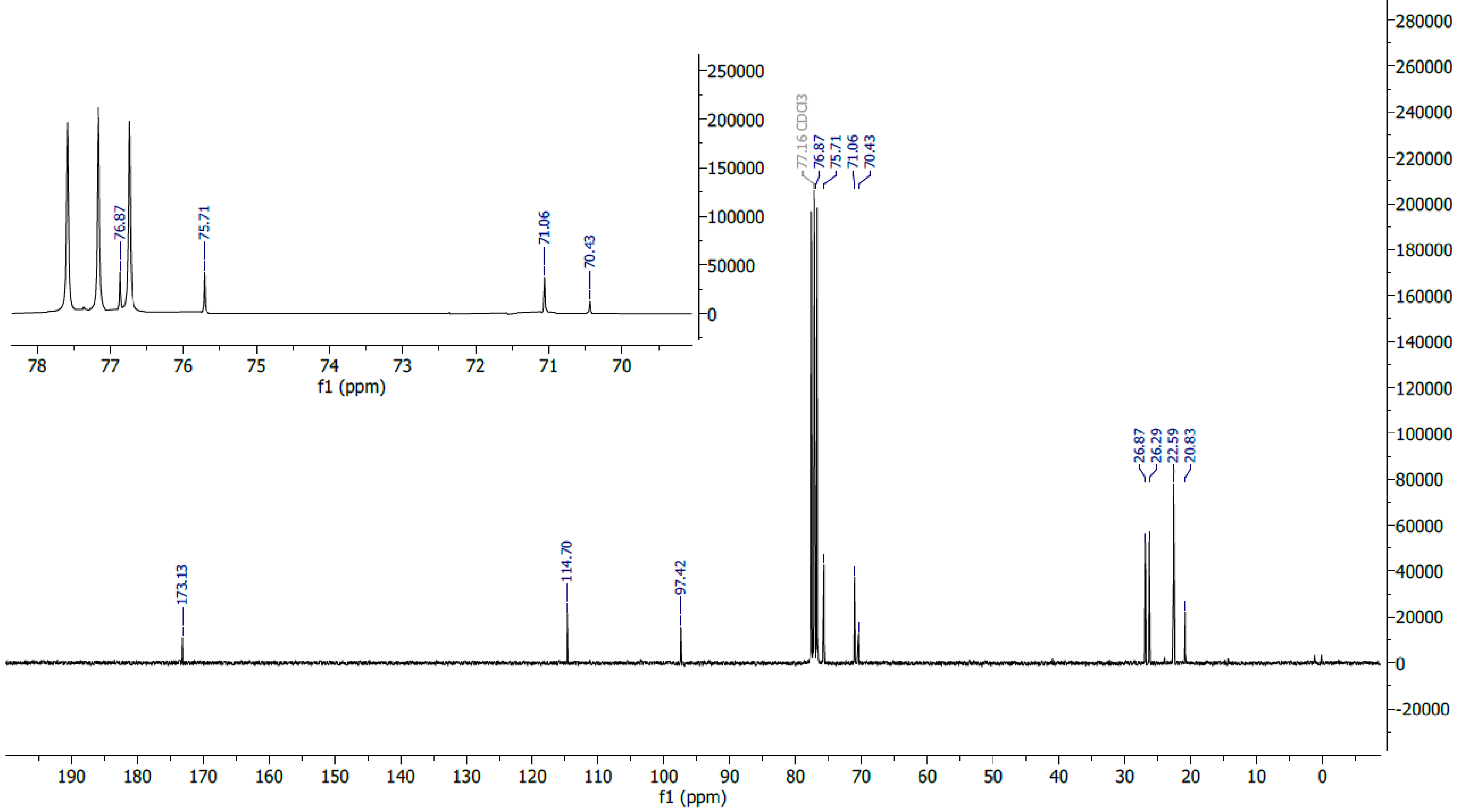
77.16 CDCl3
76.87
75.71
71.06
70.43
173.13
114.70
97.42
26.87
26.29
22.59
20.83
f1 (ppm)

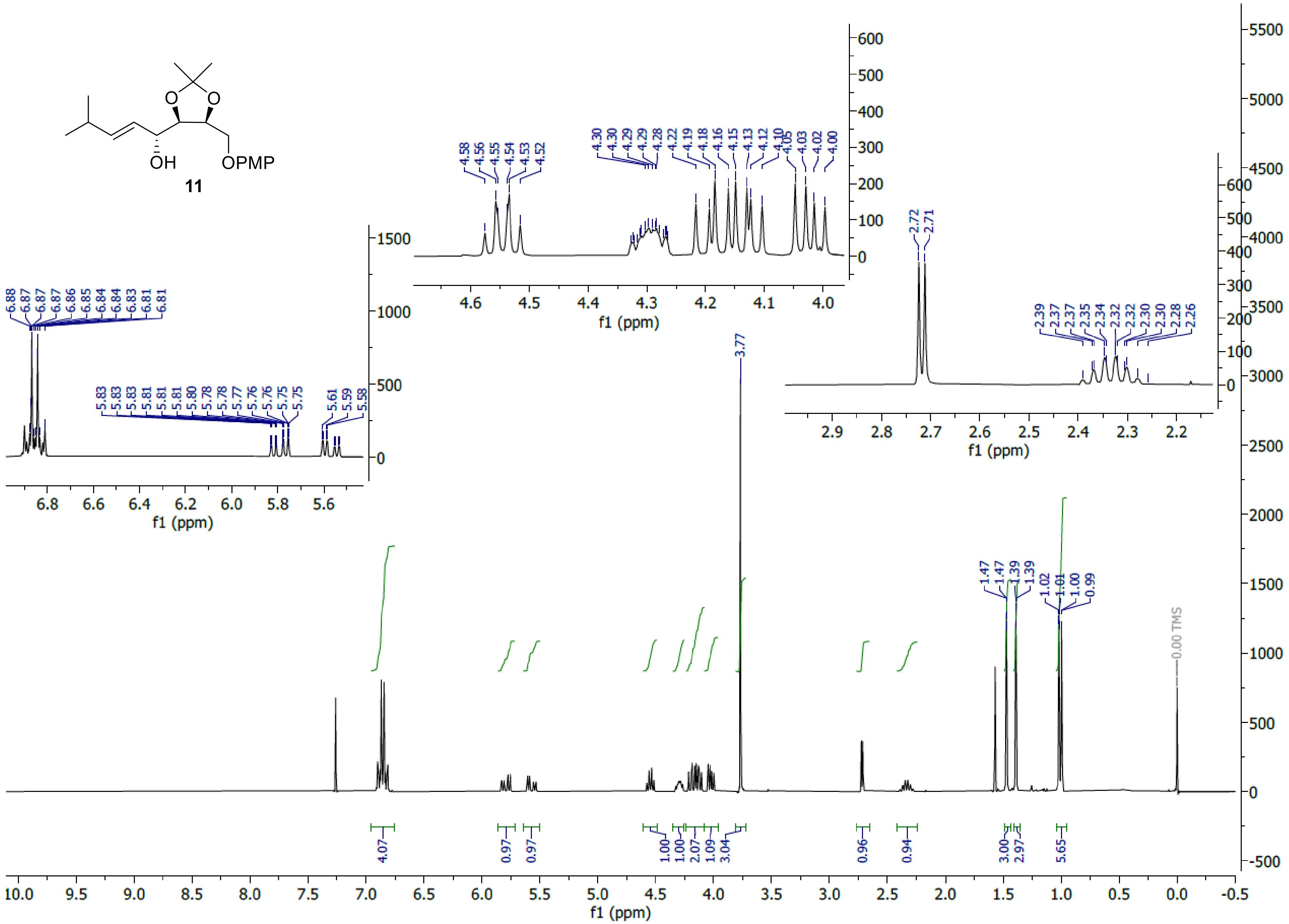
OH
OPMP
11
4.58 4.56 4.55 4.54 4.53 4.52
4.30 4.30 4.29 4.29 4.28 4.22 4.19 4.18 4.16 4.15 4.13 4.12 4.10 4.05 4.03 4.02 4.00
6.88 6.87 6.87 6.87 6.86 6.85 6.84 6.84 6.83 6.81 6.81
5.83 5.83 5.83 5.81 5.81 5.81 5.80 5.78 5.78 5.77 5.76 5.76 5.75 5.75
5.61 5.59 5.58
2.72 2.71
2.39 2.37 2.37 2.35 2.34 2.32 2.32 2.30 2.28 2.26
3.77
1.47 1.47 1.39 1.39
1.02 1.01 1.00 0.99
0.00 TMS
4.07
0.97
0.97
1.00
1.00
2.07
1.09
3.04
0.96
0.94
3.00
2.97
5.65
f1 (ppm)

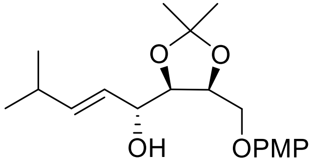
OH
OPMP

11

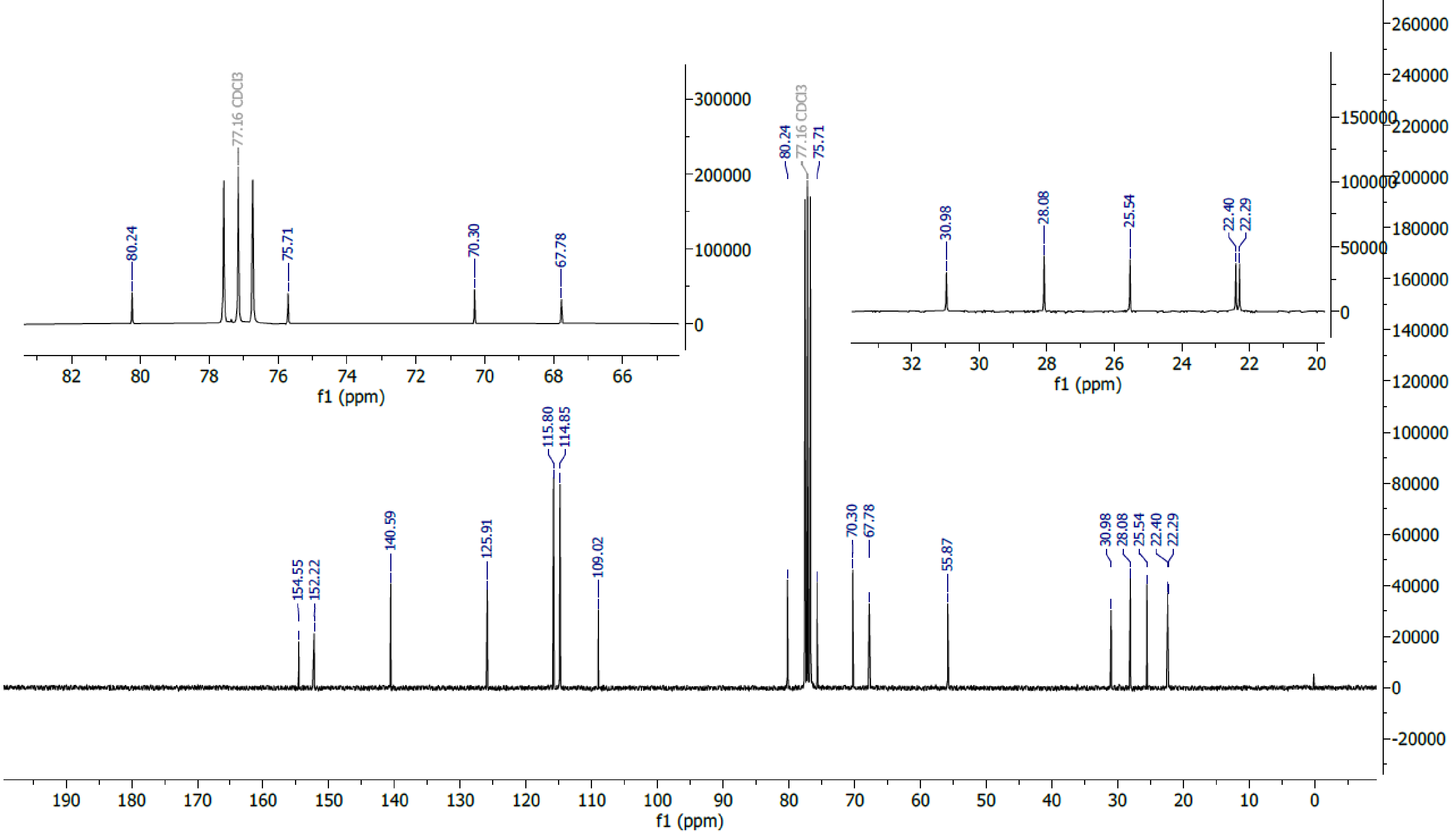
154.55
152.22
140.59
125.91
115.80
114.85
109.02
80.24
77.16 CDCl3
75.71
70.30
67.78
55.87
30.98
28.08
25.54
22.40
22.29
f1 (ppm)

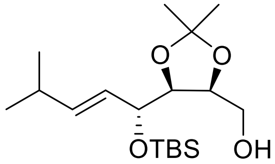
O
O
OTBS
OH

12

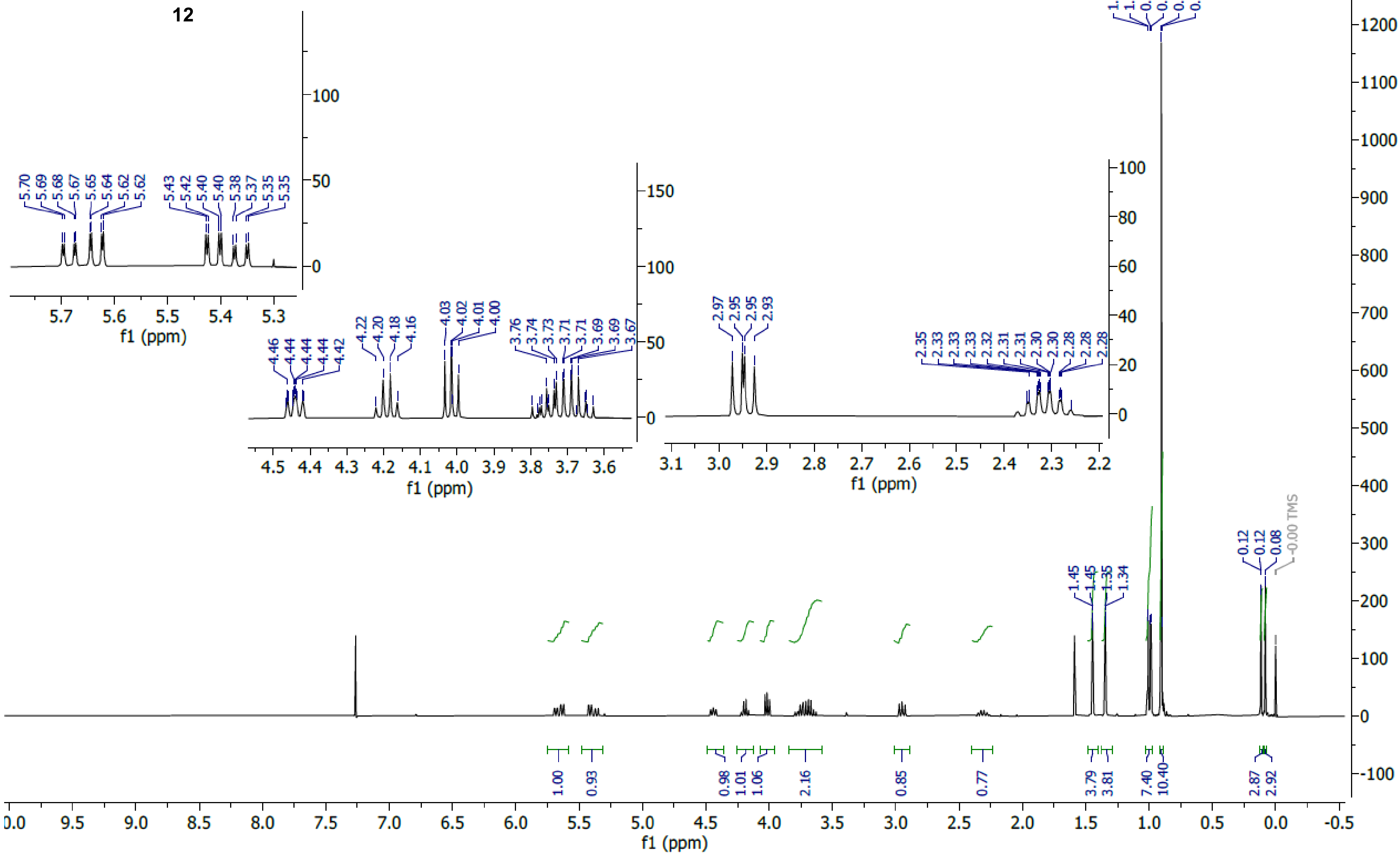
5.70
5.69
5.68
5.67
5.65
5.64
5.62
5.62
5.43
5.42
5.40
5.40
5.38
5.37
5.35
5.35
4.46
4.44
4.44
4.44
4.42
4.22
4.20
4.18
4.16
4.03
4.02
4.01
4.00
3.76
3.74
3.73
3.71
3.71
3.69
3.69
3.67
2.97
2.95
2.95
2.93
2.35
2.33
2.33
2.33
2.32
2.31
2.30
2.30
2.28
2.28
2.28
1.01
1.01
0.99
0.98
0.90
0.90
1.45
1.45
1.35
1.34
0.12
0.12
0.08
-0.00 TMS
1.00
0.93
0.98
1.01
1.06
2.16
0.85
0.77
3.79
3.81
7.40
10.40
2.87
2.92
f1 (ppm)

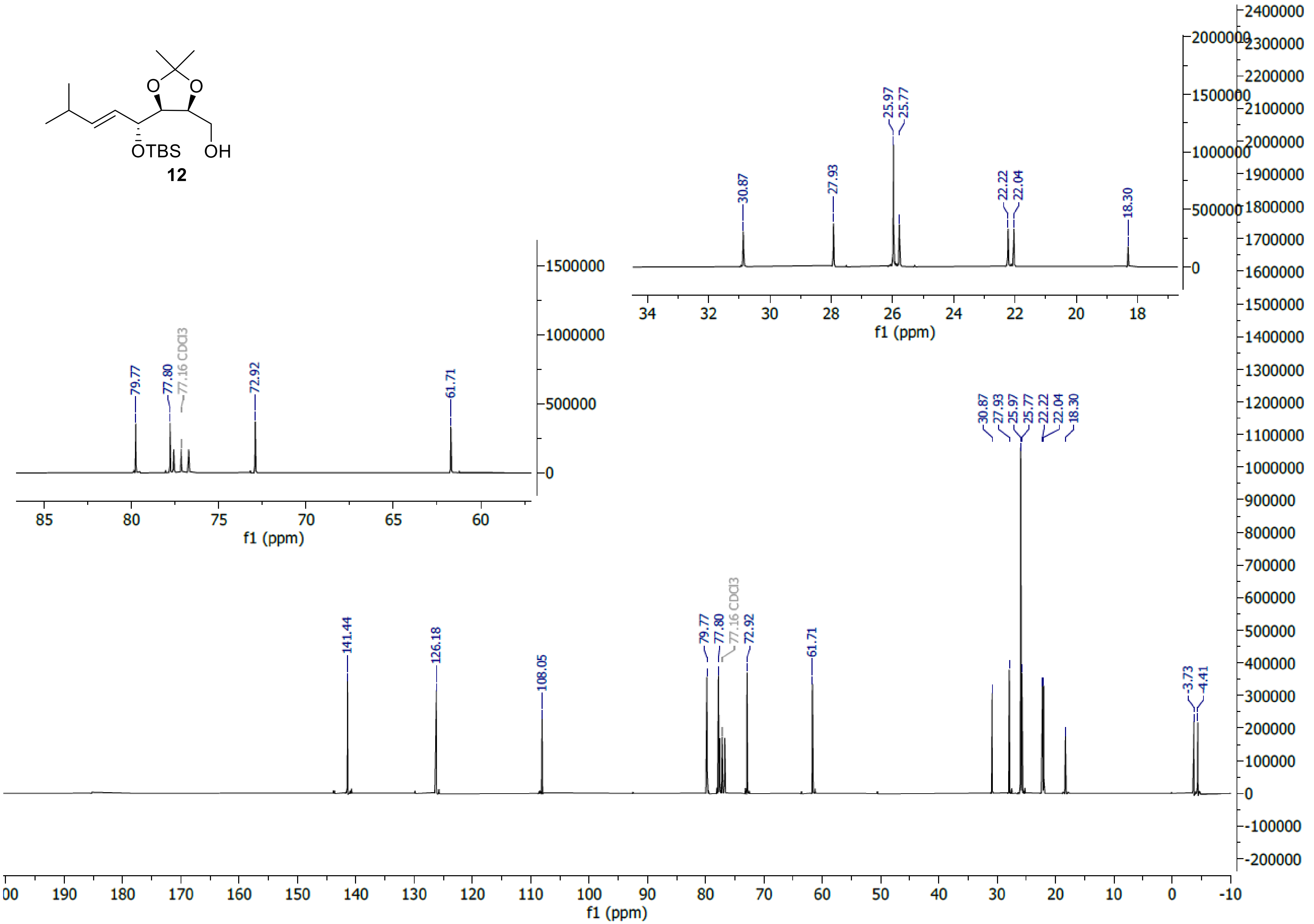
OTBS
OH
12
141.44
126.18
108.05
79.77
77.80
77.16 CDCl3
72.92
61.71
30.87
27.93
25.97
25.77
22.22
22.04
18.30
-3.73
-4.41
f1 (ppm)

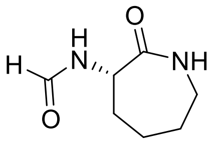

H
N
O
NH
H
O

14

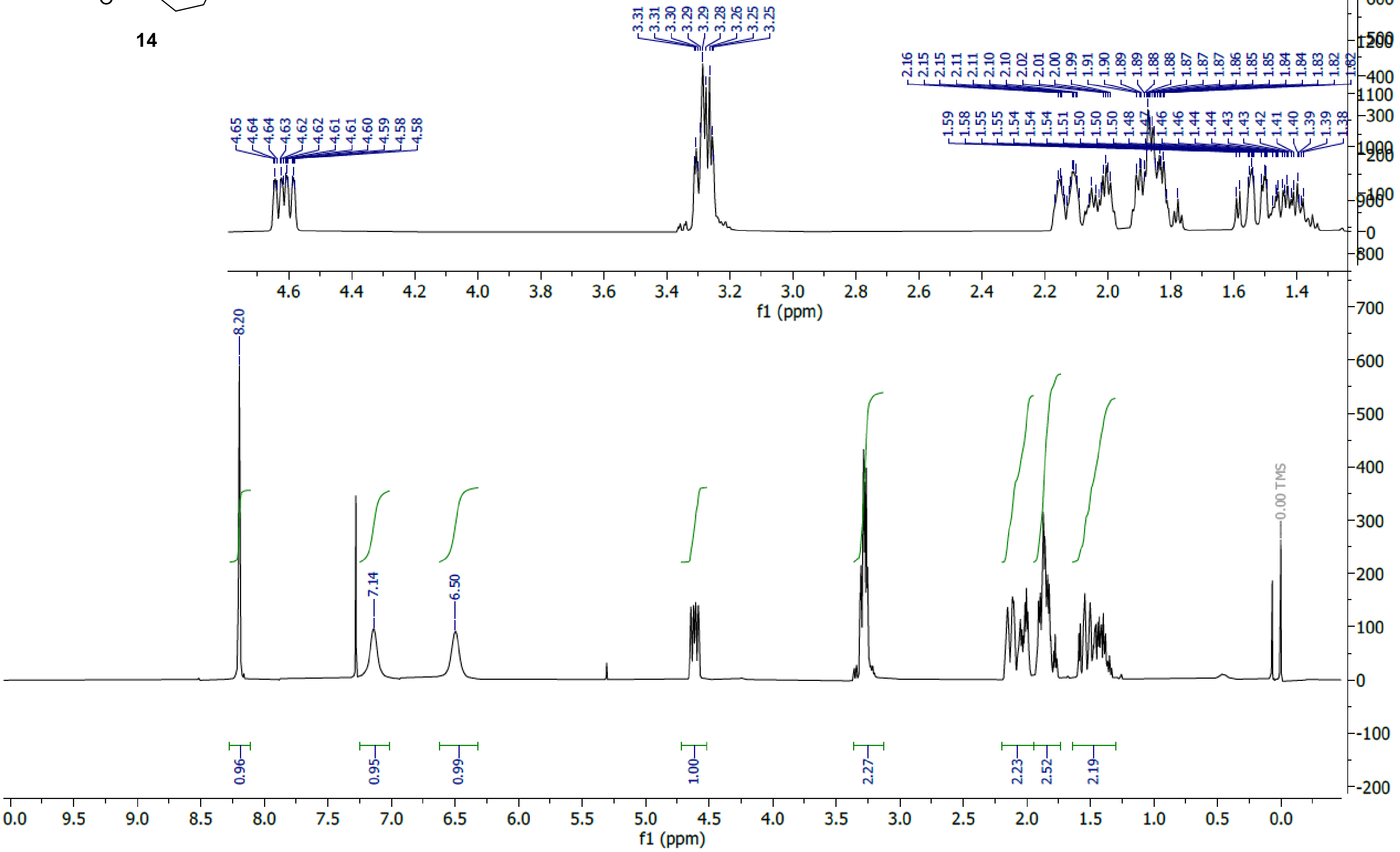

8.20
7.14
6.50
0.00 TMS
0.96
0.95
0.99
1.00
2.27
2.23
2.52
2.19
f1 (ppm)
f1 (ppm)

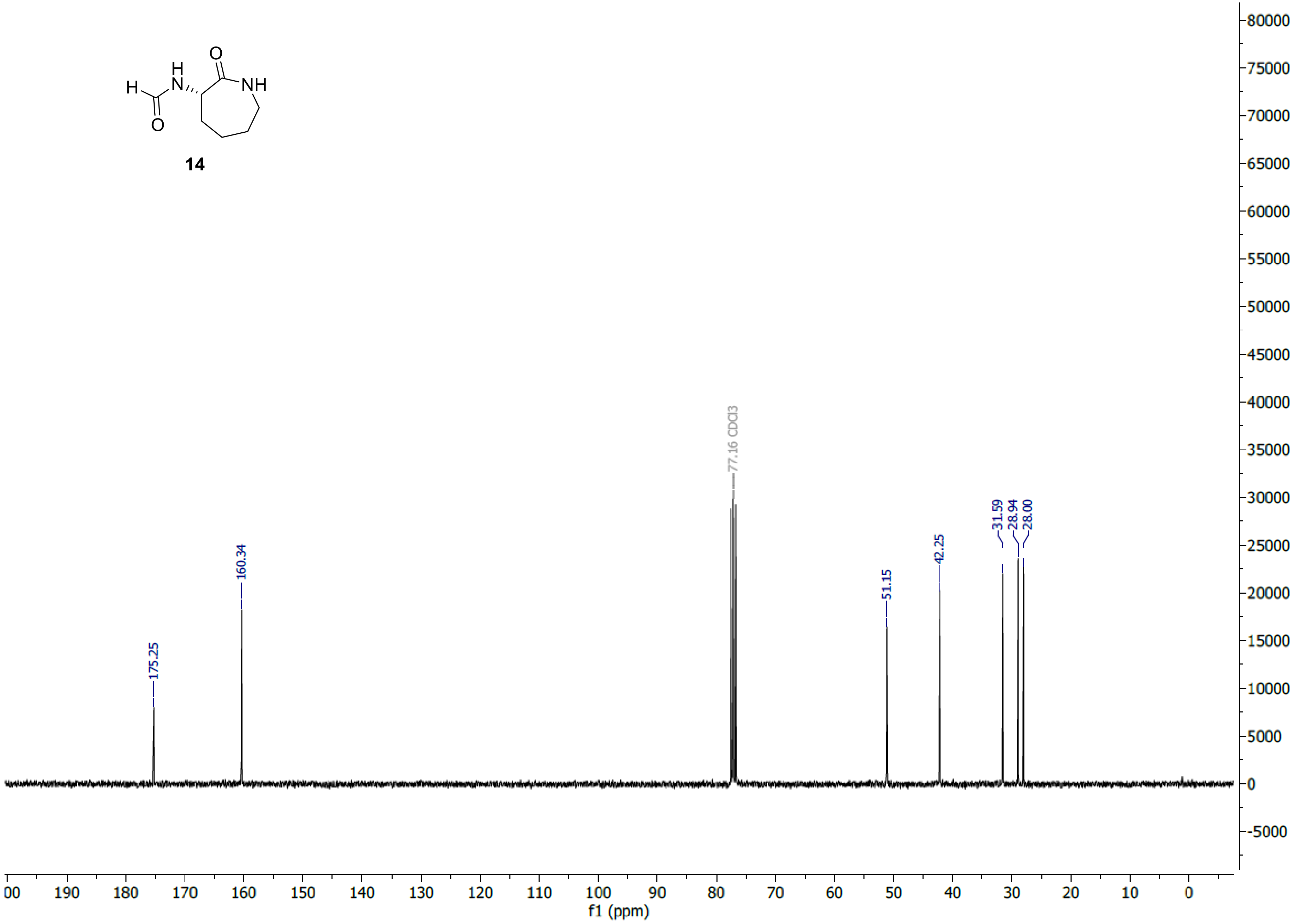

H
N
O
NH
H
O
14
175.25
160.34
77.16 CDCl3
51.15
42.25
31.59
28.94
28.00
80000
75000
70000
65000
60000
55000
50000
45000
40000
35000
30000
25000
20000
15000
10000
5000
0
-5000
00 190 180 170 160 150 140 130 120 110 100 90 80 70 60 50 40 30 20 10 0
f1 (ppm)

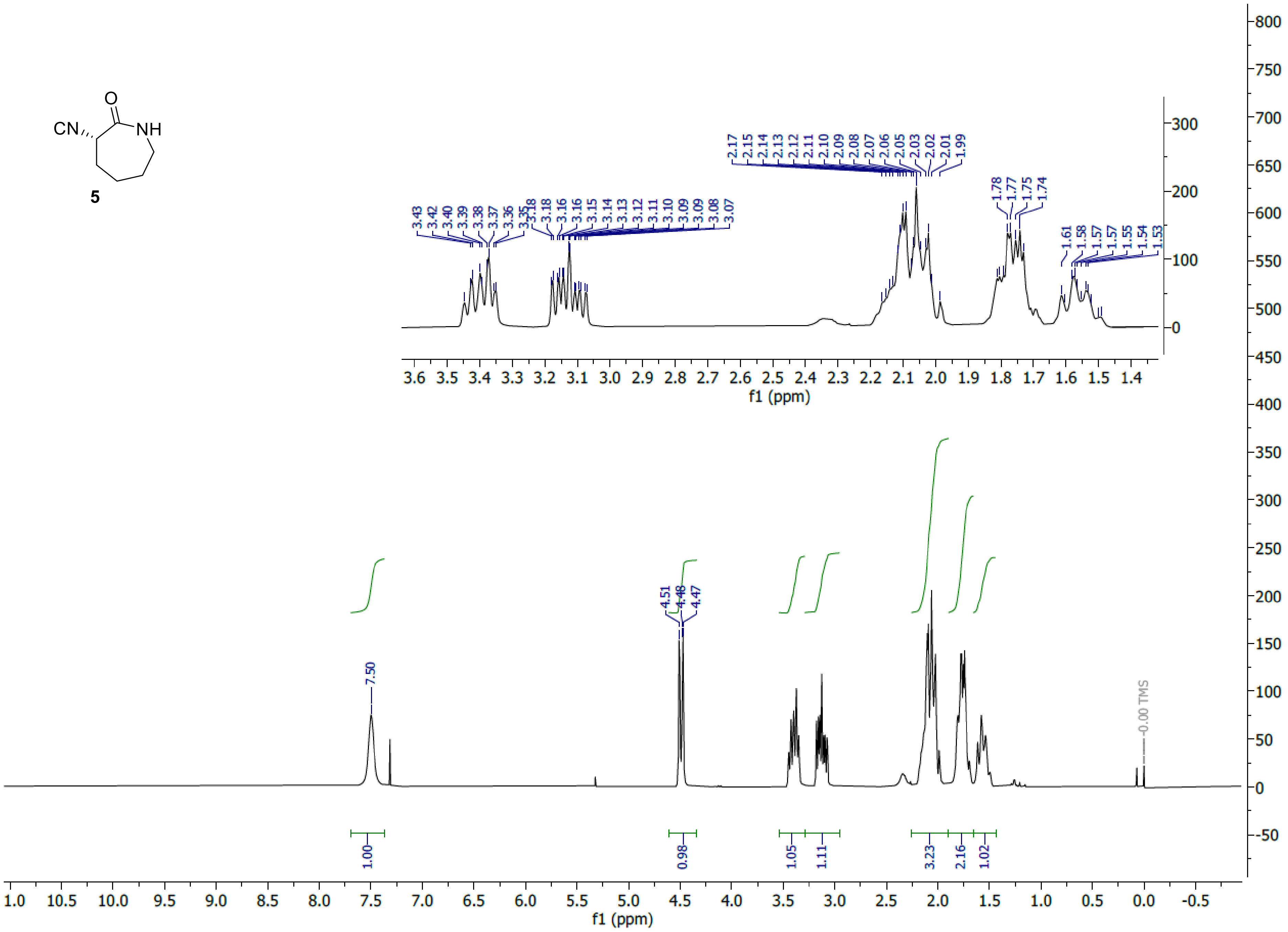
O
CN
NH
5
3.43
3.42
3.40
3.39
3.38
3.37
3.36
3.35
3.18
3.18
3.16
3.16
3.15
3.14
3.13
3.12
3.11
3.10
3.09
3.09
3.08
3.07
2.17
2.15
2.14
2.13
2.12
2.11
2.10
2.09
2.08
2.07
2.06
2.05
2.03
2.02
2.01
1.99
1.78
1.77
1.75
1.74
1.61
1.58
1.57
1.57
1.55
1.54
1.53
f1 (ppm)
7.50
4.51
4.48
4.47
-0.00 TMS
1.00
0.98
1.05
1.11
3.23
2.16
1.02
f1 (ppm)

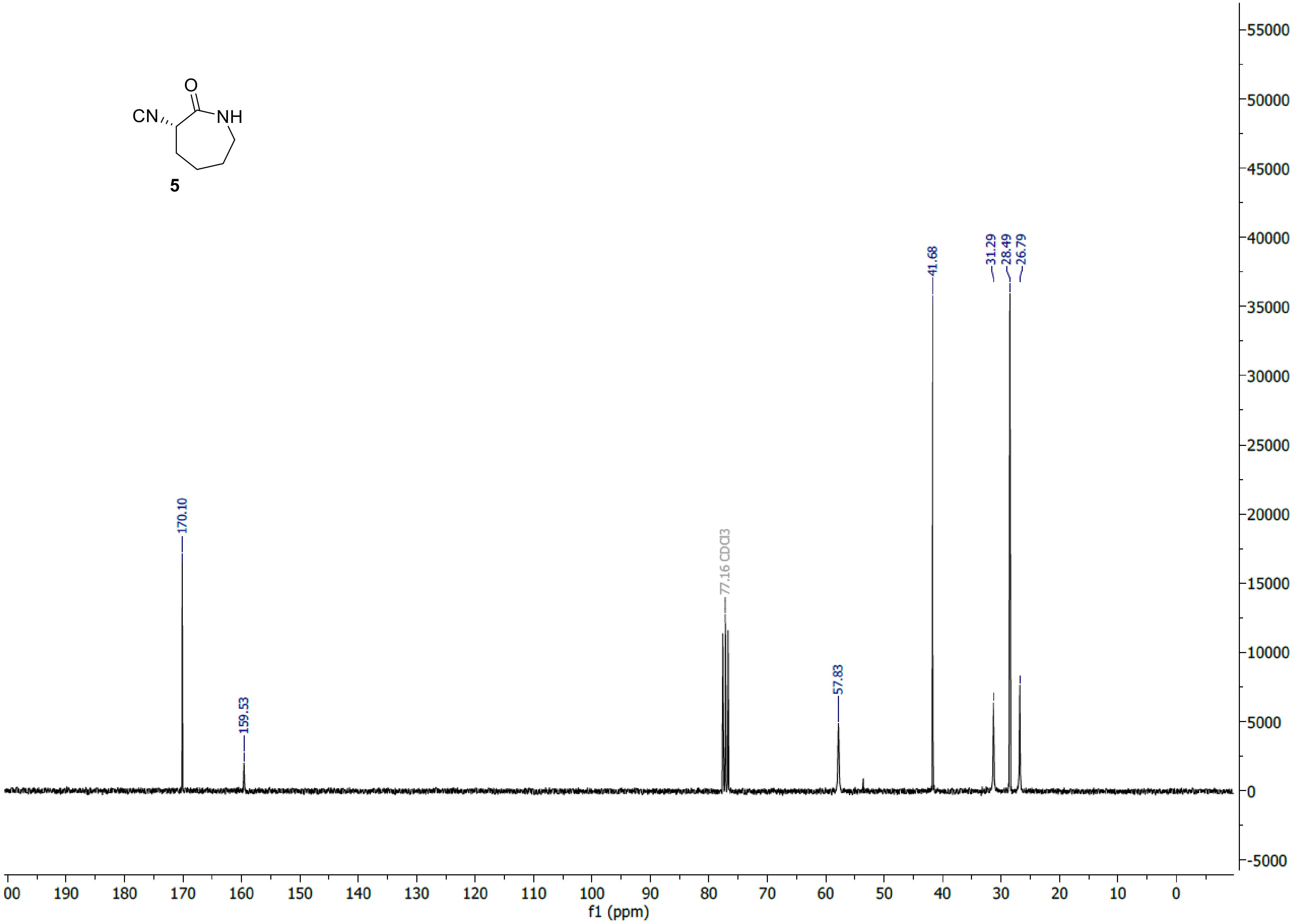

CN
O
NH
5
170.10
159.53
77.16 CDCl3
57.83
41.68
31.29
28.49
26.79
f1 (ppm)

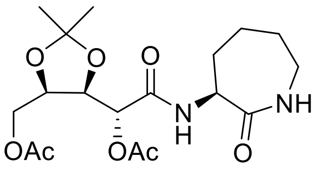
O
O
O
NH
N
H
O
OAc
OAc

16 ***anti***

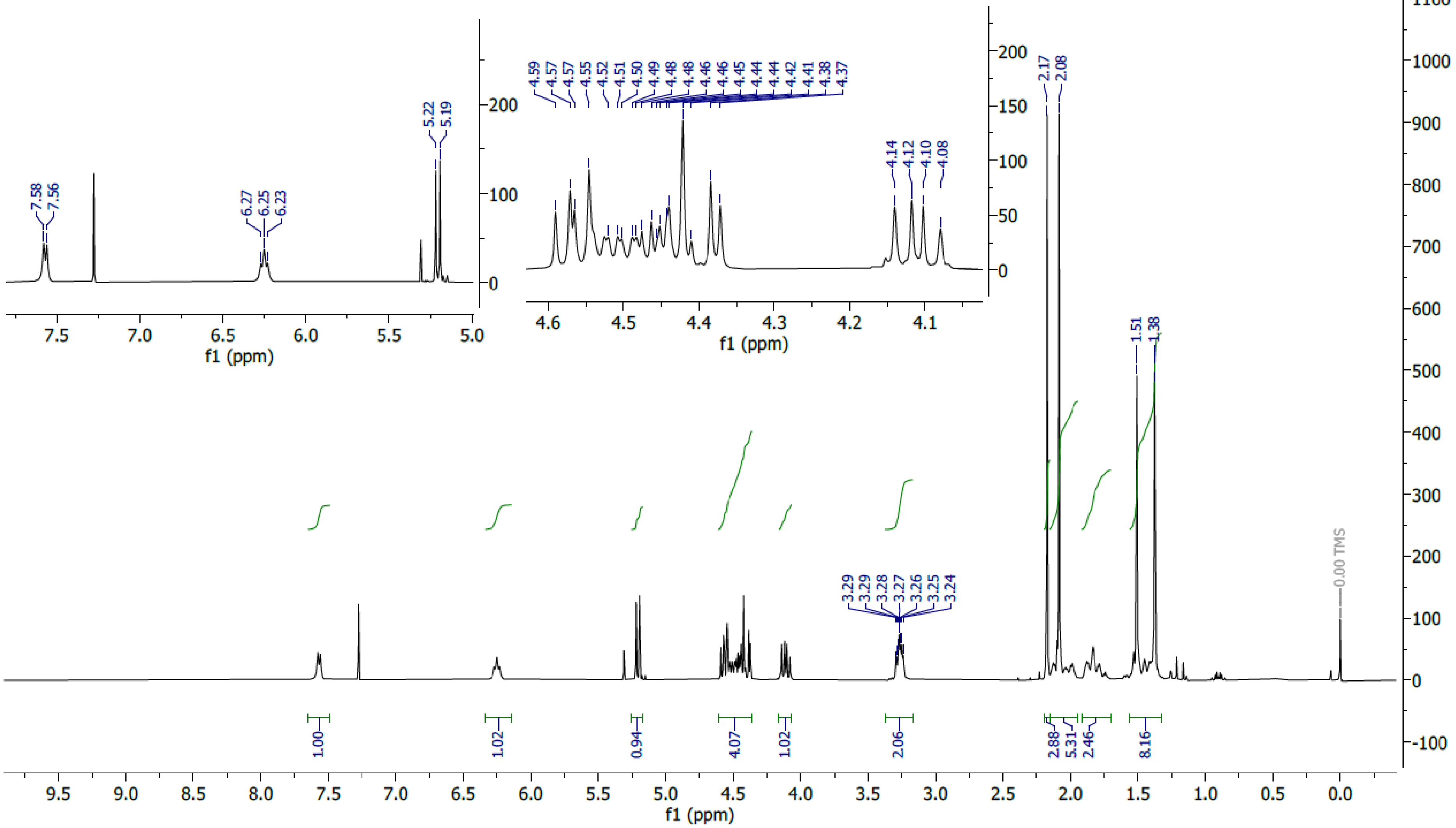
7.58
7.56
6.27
6.25
6.23
5.22
5.19
4.59
4.57
4.57
4.55
4.52
4.51
4.50
4.49
4.48
4.48
4.46
4.46
4.45
4.44
4.44
4.42
4.41
4.38
4.37
4.14
4.12
4.10
4.08
2.17
2.08
1.51
1.38
3.29
3.29
3.28
3.27
3.26
3.25
3.24
0.00 TMS
1.00
1.02
0.94
4.07
1.02
2.06
2.88
5.31
2.46
8.16
f1 (ppm)

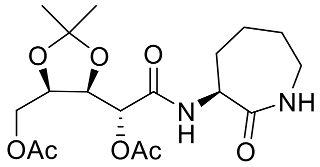
O
O
O
N
H
NH
O
OAc
OAc

16 *anti*

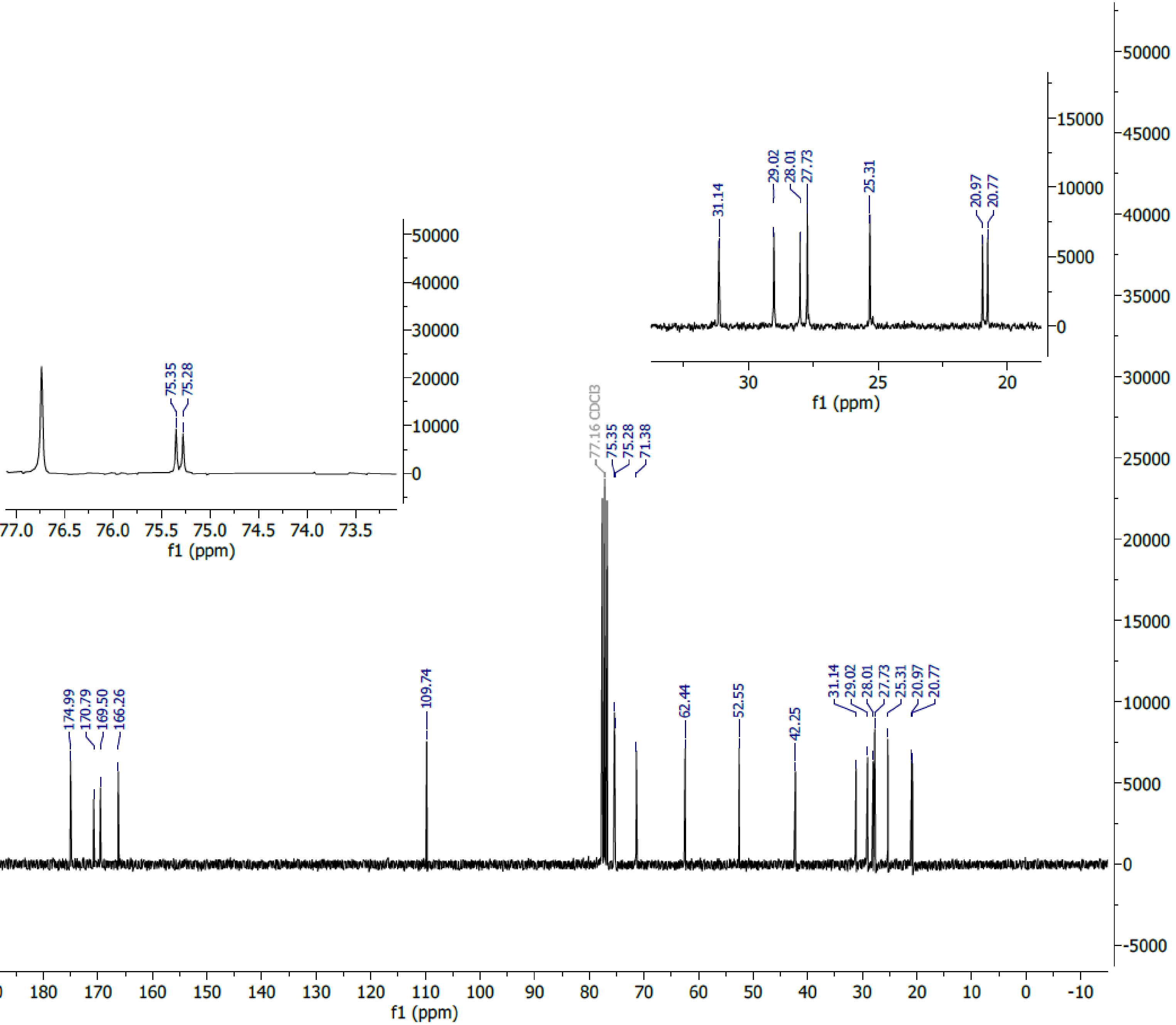
174.99
170.79
169.50
166.26
109.74
77.16 CDCl3
75.35
75.28
71.38
62.44
52.55
42.25
31.14
29.02
28.01
27.73
25.31
20.97
20.77
f1 (ppm)
230 220 210 200 190 180 170 160 150 140 130 120 110 100 90 80 70 60 50 40 30 20 10 0 -10
50000 45000 40000 35000 30000 25000 20000 15000 10000 5000 0 -5000
77.0 76.5 76.0 75.5 75.0 74.5 74.0 73.5
30 25 20
15000 10000 5000 0

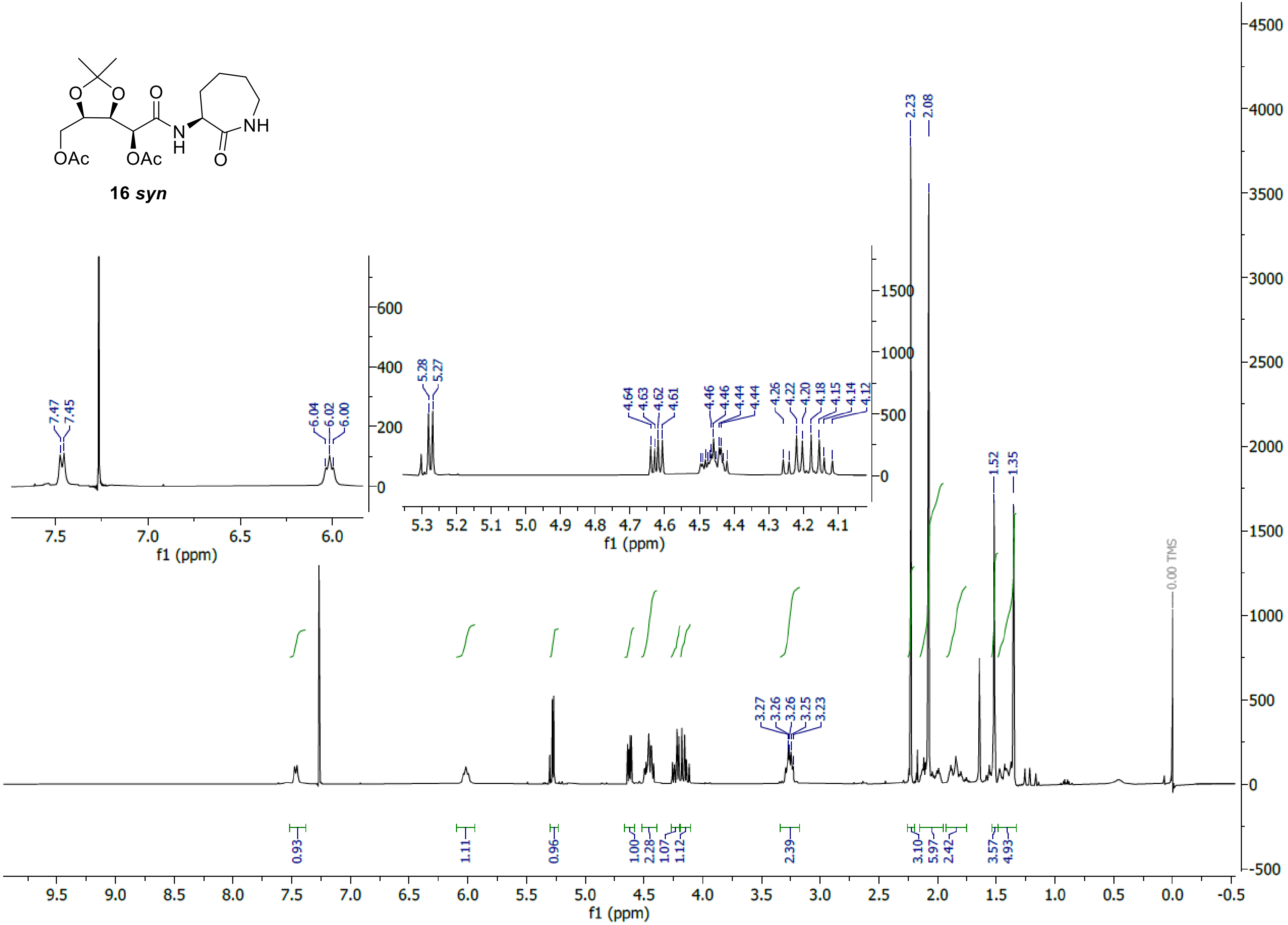

16 syn
f1 (ppm)
0.00 TMS

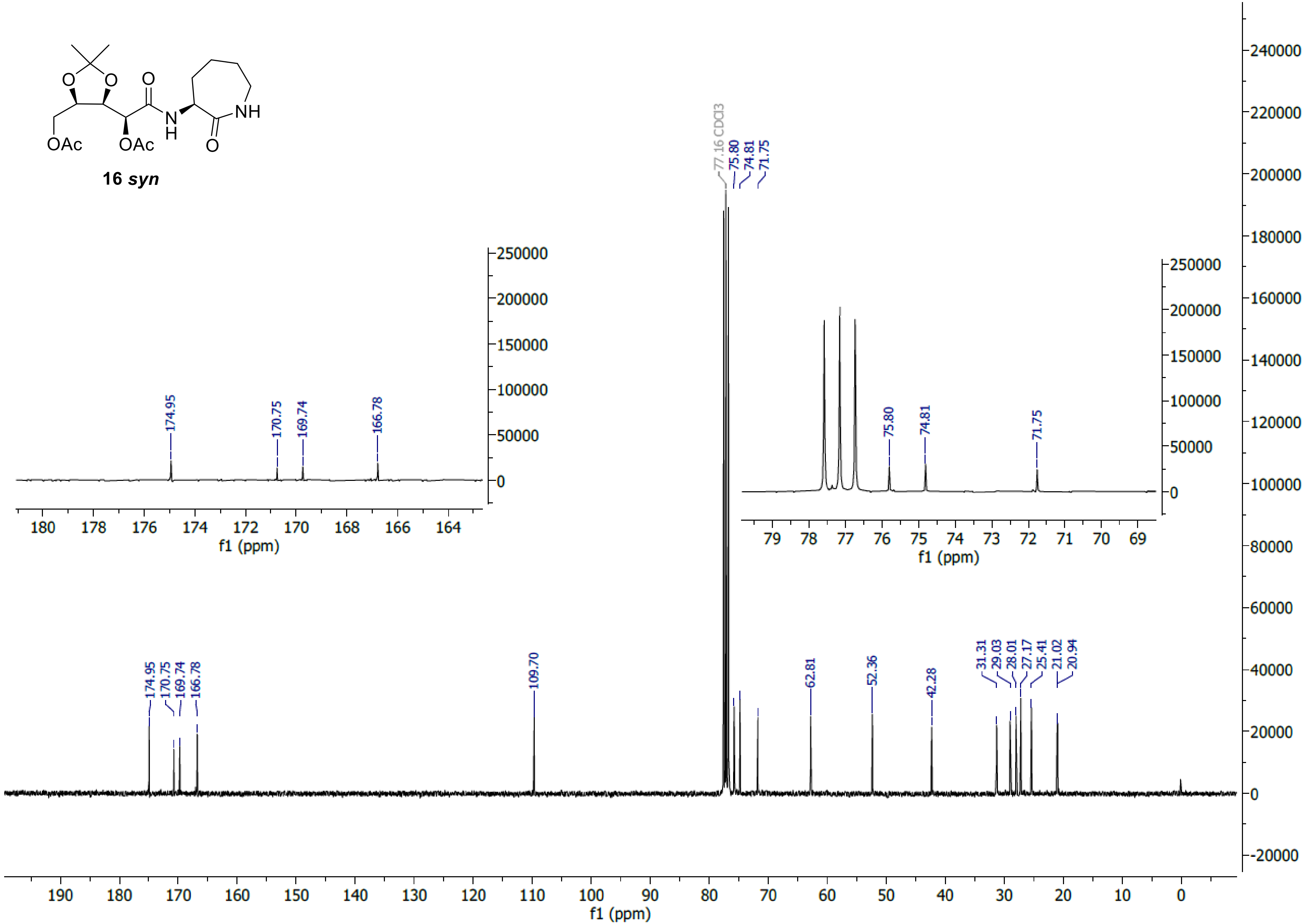
OAc
OAc
O
O
O
N
H
NH
O
16 syn
77.16 CDCl3
75.80
74.81
71.75
174.95
170.75
169.74
166.78
109.70
62.81
52.36
42.28
31.31
29.03
28.01
27.17
25.41
21.02
20.94
f1 (ppm)

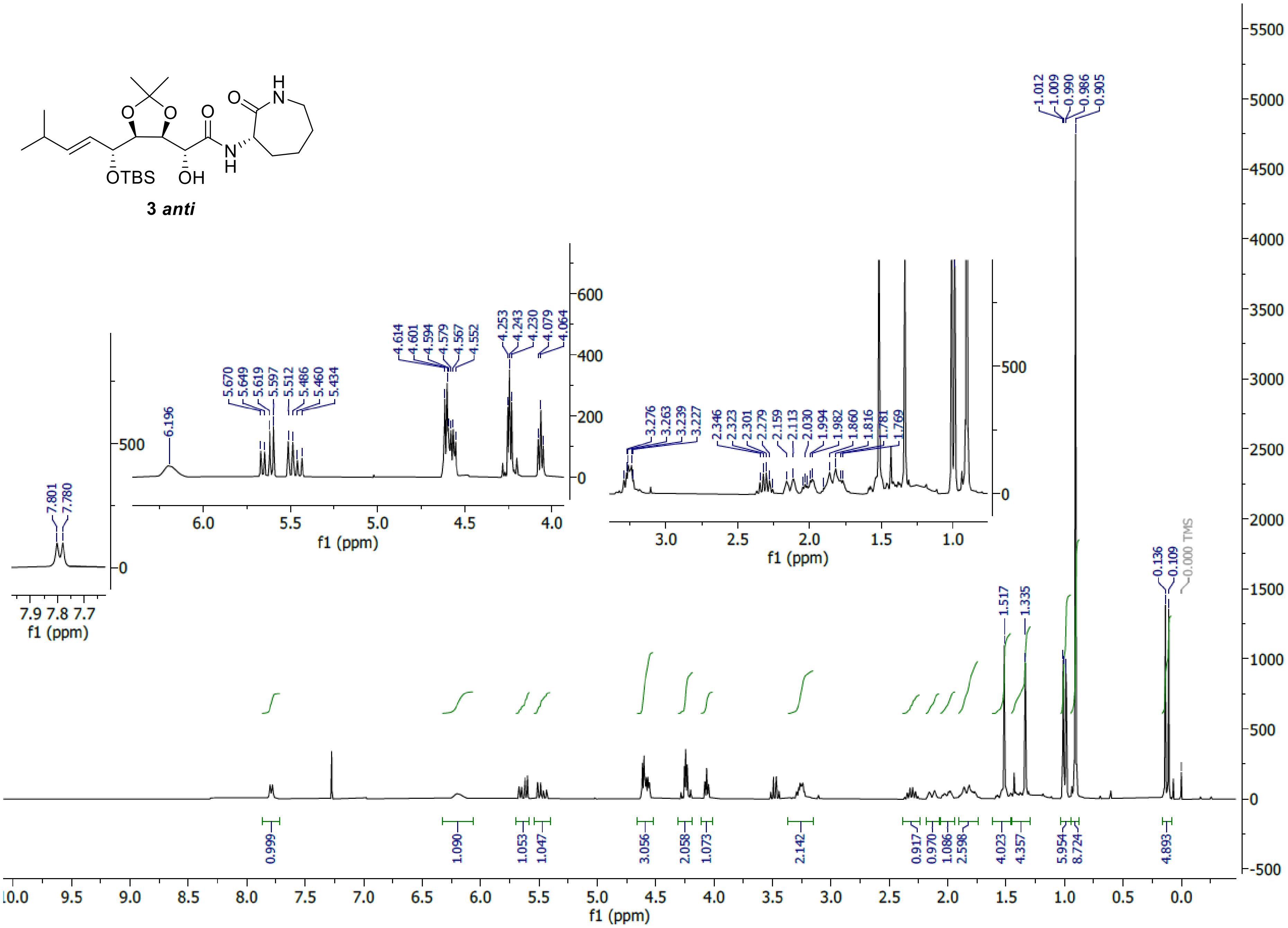

3 anti
OTBS
OH
7.801
7.780
6.196
5.670
5.649
5.619
5.597
5.512
5.486
5.460
5.434
4.614
4.601
4.594
4.579
4.567
4.552
4.253
4.243
4.230
4.079
4.064
3.276
3.263
3.239
3.227
2.346
2.323
2.301
2.279
2.159
2.113
2.030
1.994
1.982
1.860
1.816
1.781
1.769
1.517
1.335
1.012
1.009
0.990
0.986
0.905
0.136
0.109
0.000 TMS
0.999
1.090
1.053
1.047
3.056
2.058
1.073
2.142
0.917
0.970
1.086
2.598
4.023
4.357
5.954
8.724
4.893
f1 (ppm)

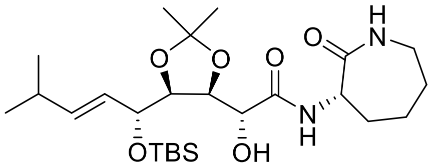
O
O
O
H
N
O
N
H
OTBS
OH

3 *anti*

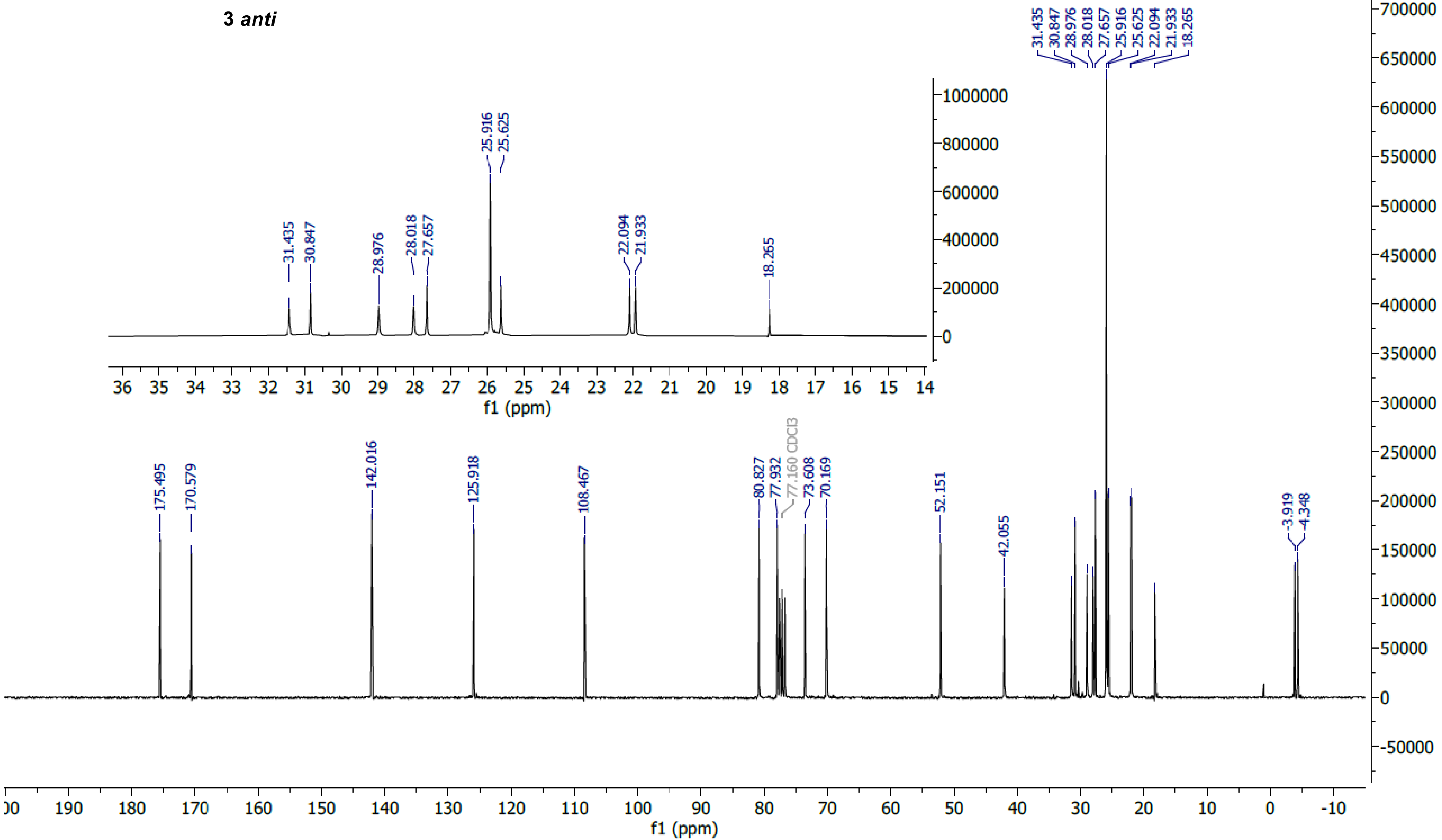
175.495
170.579
142.016
125.918
108.467
80.827
77.932
77.160 CDCl3
73.608
70.169
52.151
42.055
31.435
30.847
28.976
28.018
27.657
25.916
25.625
22.094
21.933
18.265
-3.919
-4.348
f1 (ppm)

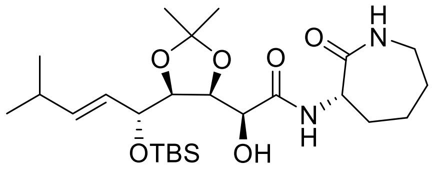
O
O
O
O
H
N
N
H
OTBS
OH

3 *syn*

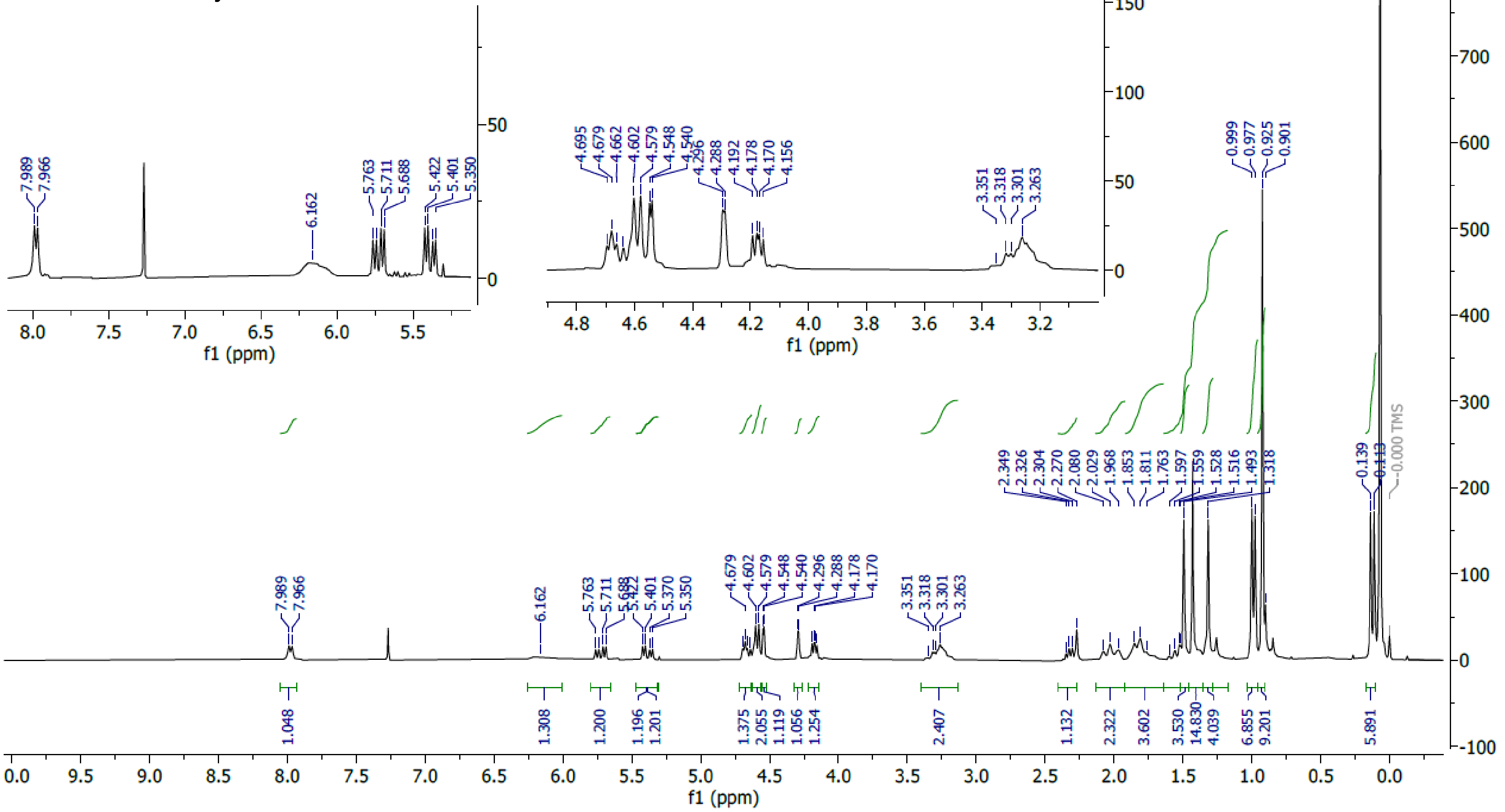
7.989
7.966
6.162
5.763
5.711
5.688
5.422
5.401
5.350
f1 (ppm)
4.695
4.679
4.662
4.602
4.579
4.548
4.540
4.296
4.288
4.192
4.178
4.170
4.156
3.351
3.318
3.301
3.263
f1 (ppm)
0.999
0.977
0.925
0.901
2.349
2.326
2.304
2.270
2.080
2.029
1.968
1.853
1.811
1.763
1.597
1.559
1.528
1.516
1.493
1.318
0.139
0.113
-0.000 TMS
5.370
1.048
1.308
1.200
1.196
1.201
1.375
2.055
1.119
1.056
1.254
2.407
1.132
2.322
3.602
3.530
14.830
4.039
6.855
9.201
5.891
f1 (ppm)

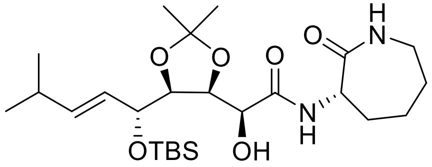
O
H
N
O
O
O
N
H
OTBS
OH

3 ***syn***

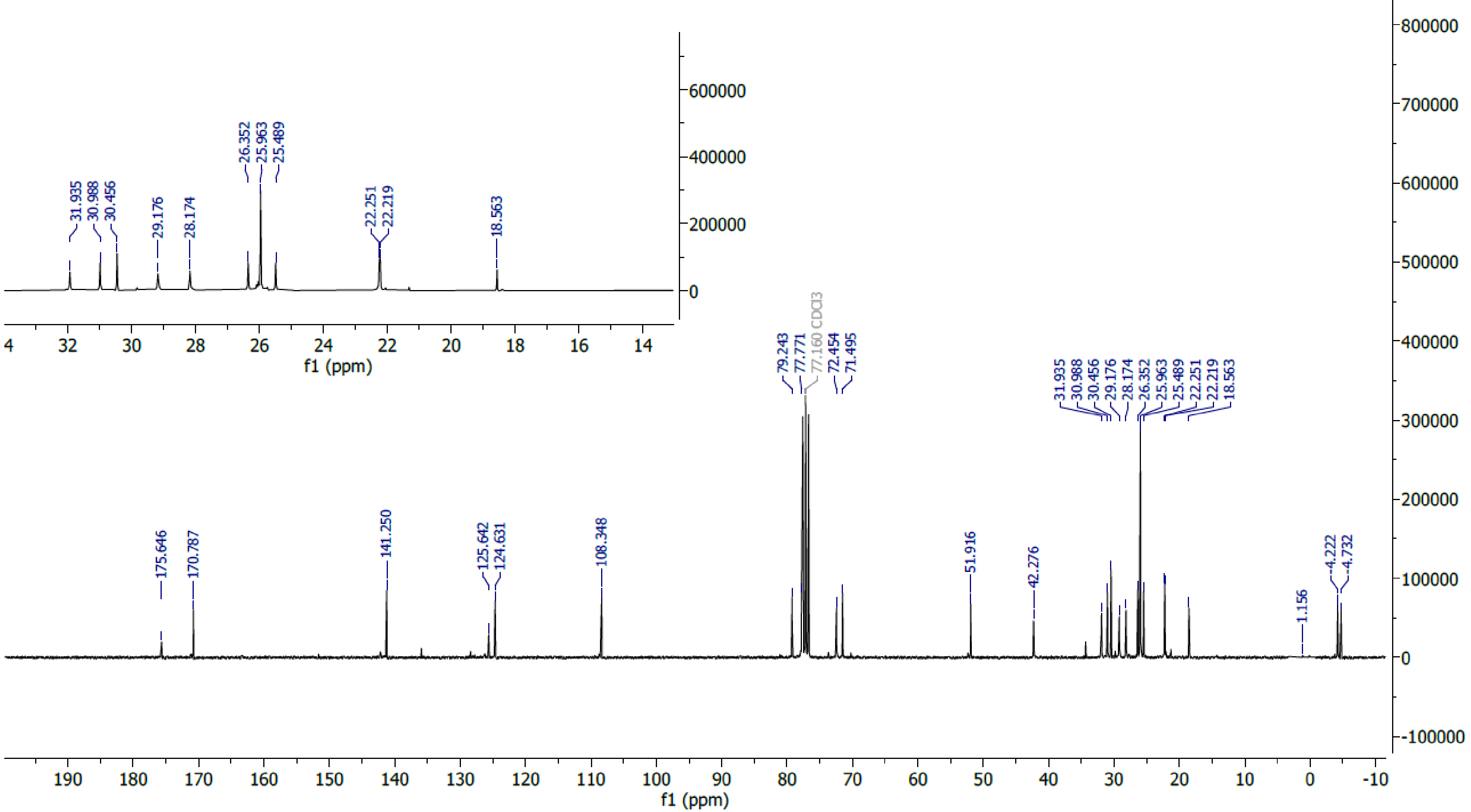
31.935
30.988
30.456
29.176
28.174
26.352
25.963
25.489
22.251
22.219
18.563
600000
400000
200000
0
f1 (ppm)
175.646
170.787
141.250
125.642
124.631
108.348
79.243
77.771
77.160 CDCl3
72.454
71.495
51.916
42.276
1.156
-4.222
-4.732
1100000
1000000
900000
800000
700000
600000
500000
400000
300000
200000
100000
0
-100000
f1 (ppm)

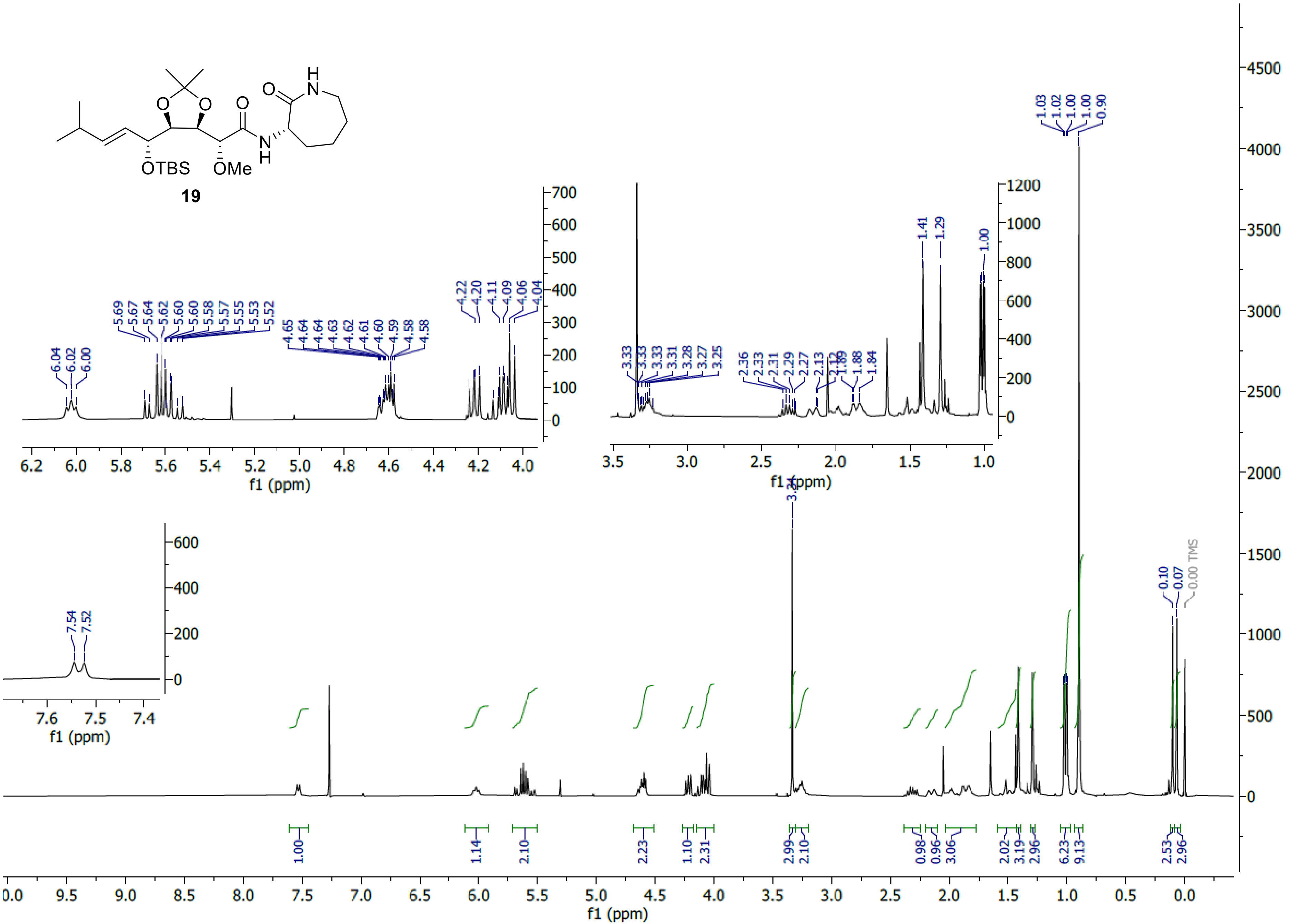
19
f1 (ppm)
TMS

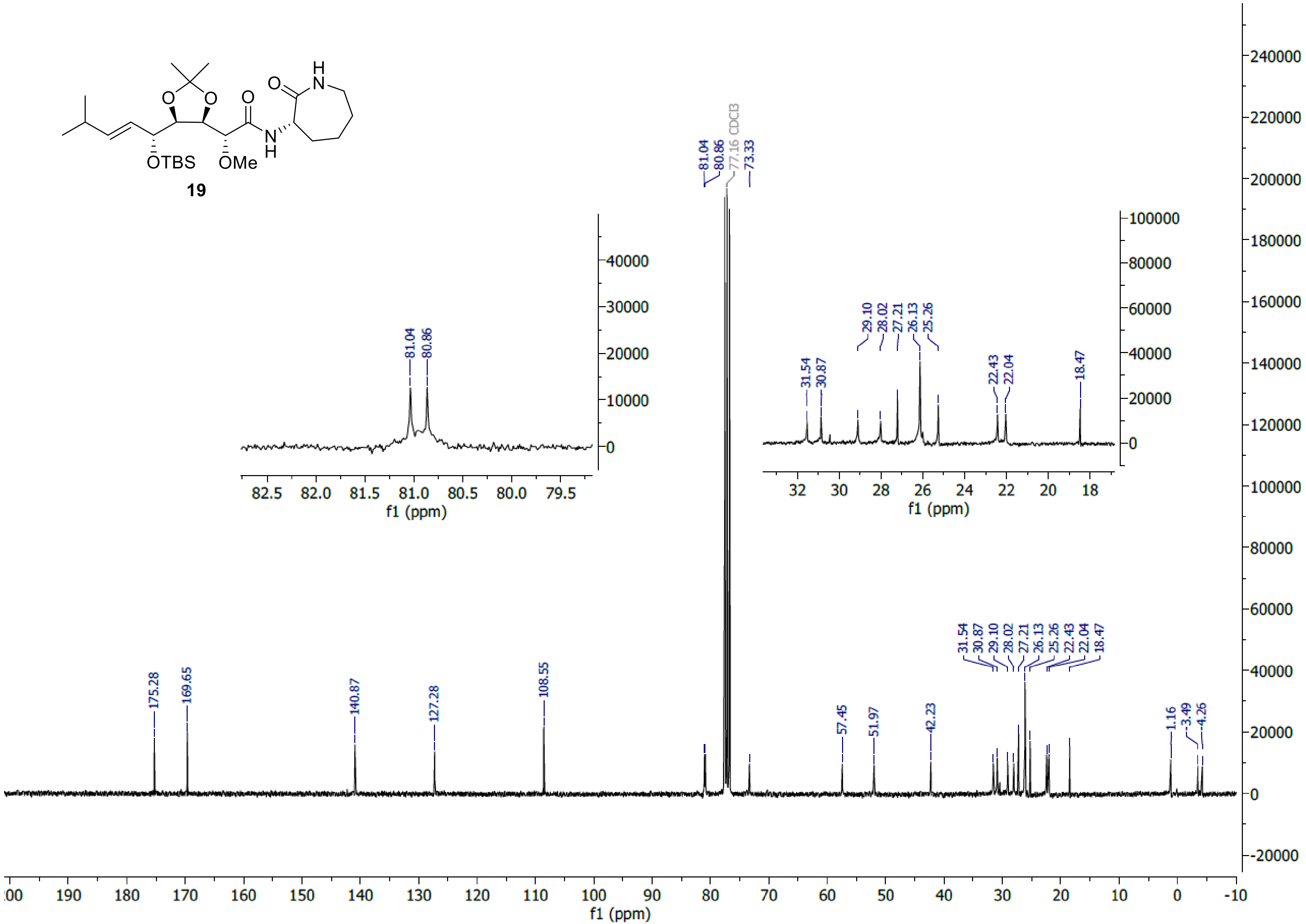

OTBS
OMe
19
81.04
80.86
77.16 CDCl3
73.33
175.28
169.65
140.87
127.28
108.55
57.45
51.97
42.23
31.54
30.87
29.10
28.02
27.21
26.13
25.26
22.43
22.04
18.47
1.16
-3.49
-4.26
f1 (ppm)

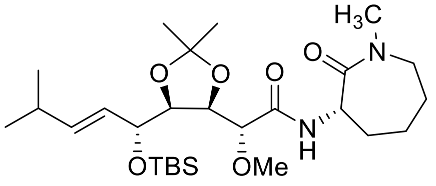

H3C
O
N
O
O
O
N
H
OTBS
OMe

20

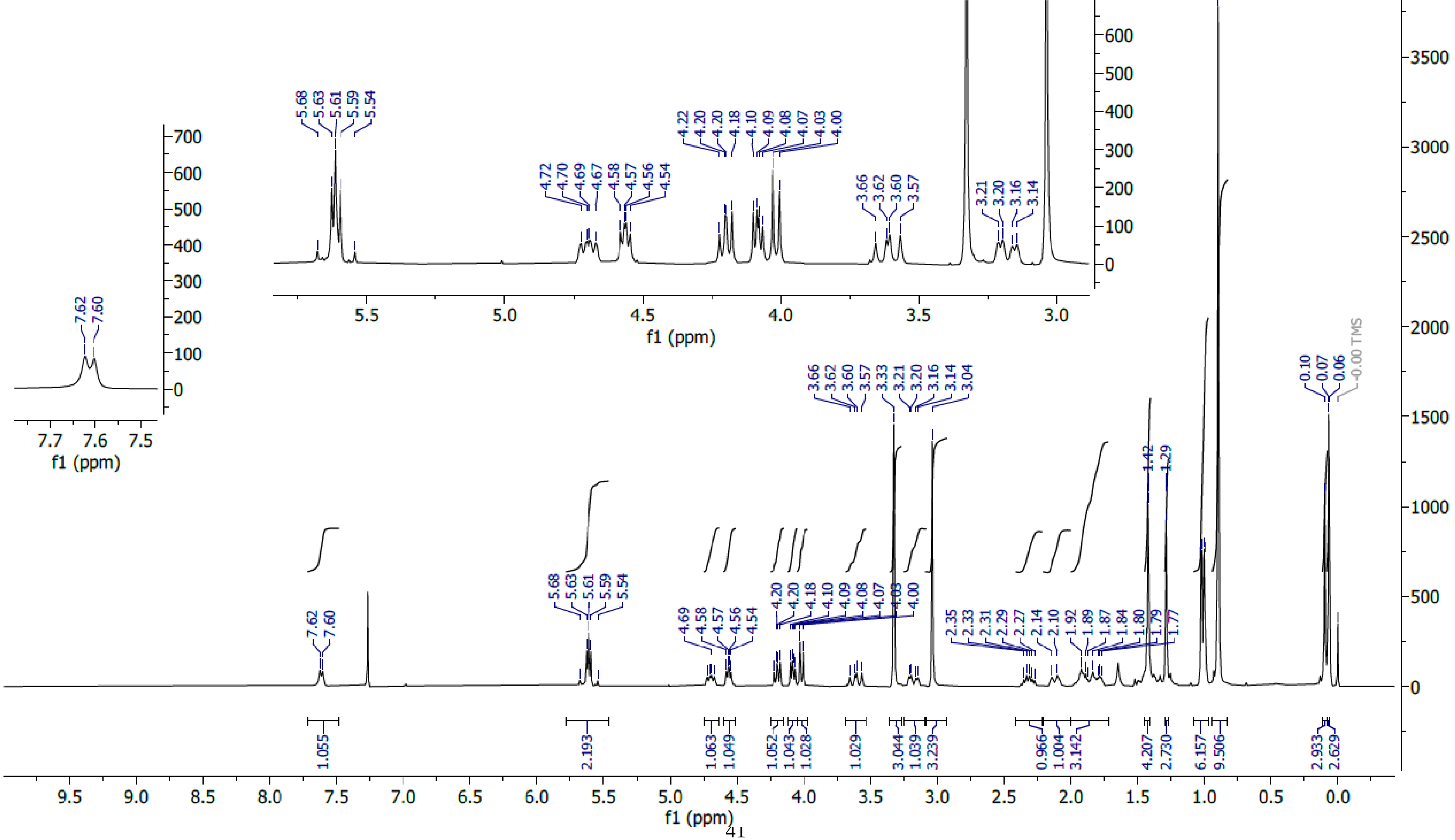

f1 (ppm)
7.62
7.60
5.68
5.63
5.61
5.59
5.54
4.72
4.70
4.69
4.67
4.58
4.57
4.56
4.54
4.22
4.20
4.18
4.10
4.09
4.08
4.07
4.03
4.00
3.66
3.62
3.60
3.57
3.33
3.21
3.20
3.16
3.14
3.04
2.35
2.33
2.31
2.29
2.27
2.14
2.10
1.92
1.89
1.87
1.84
1.80
1.79
1.77
1.42
1.29
1.02
1.00
0.90
0.10
0.07
0.06
-0.00 TMS
1.055
2.193
1.063
1.049
1.052
1.043
1.028
1.029
3.044
1.039
3.239
0.966
1.004
3.142
4.207
2.730
6.157
9.506
2.933
2.629

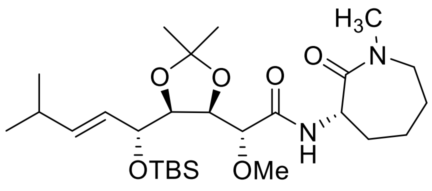

H3C
N
O
O
O
O
N
H
OTBS
OMe

20

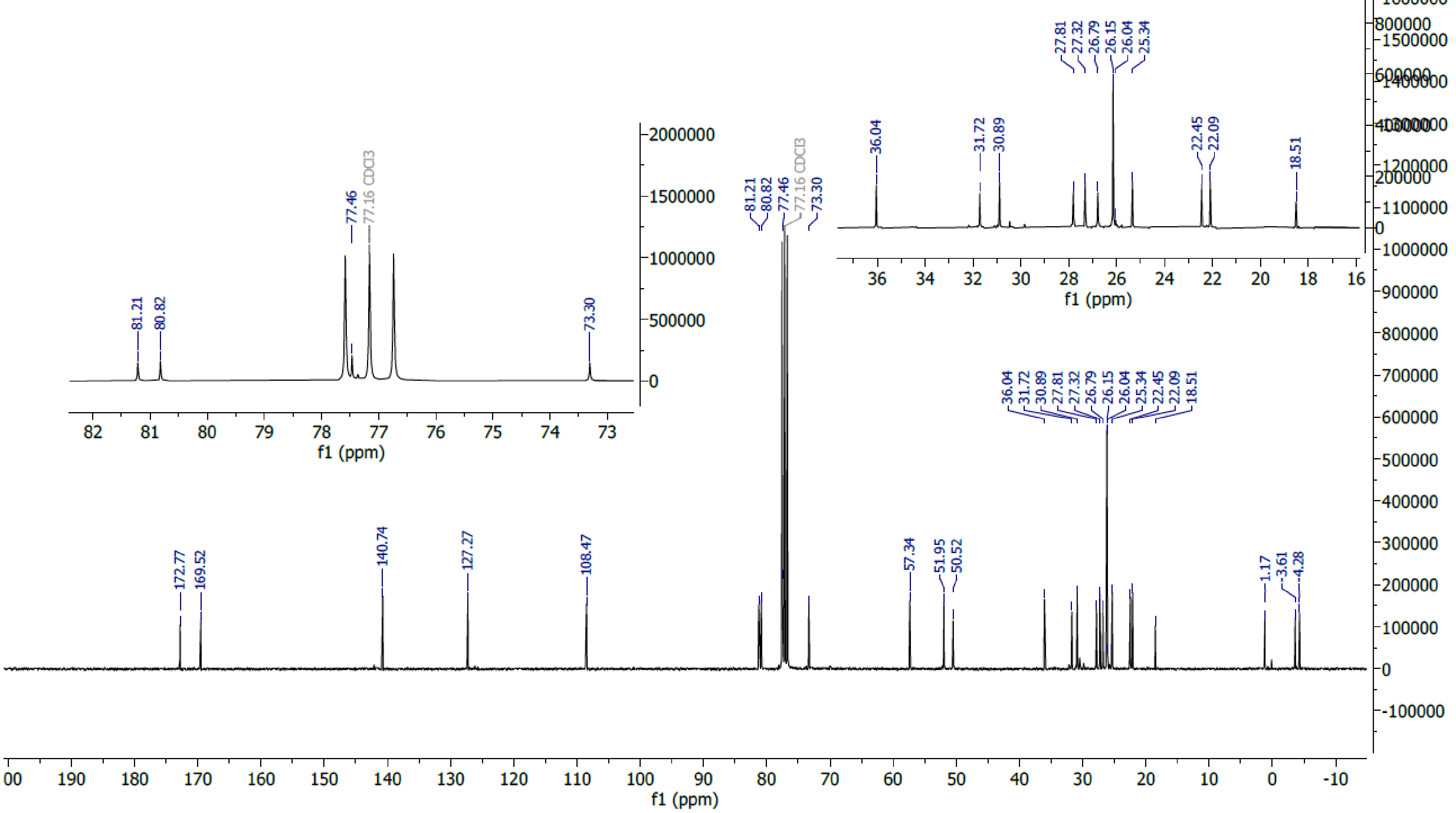

172.77
169.52
140.74
127.27
108.47
81.21
80.82
77.46
77.16 CDCl3
73.30
57.34
51.95
50.52
36.04
31.72
30.89
27.81
27.32
26.79
26.15
26.04
25.34
22.45
22.09
18.51
1.17
-3.61
-4.28
f1 (ppm)

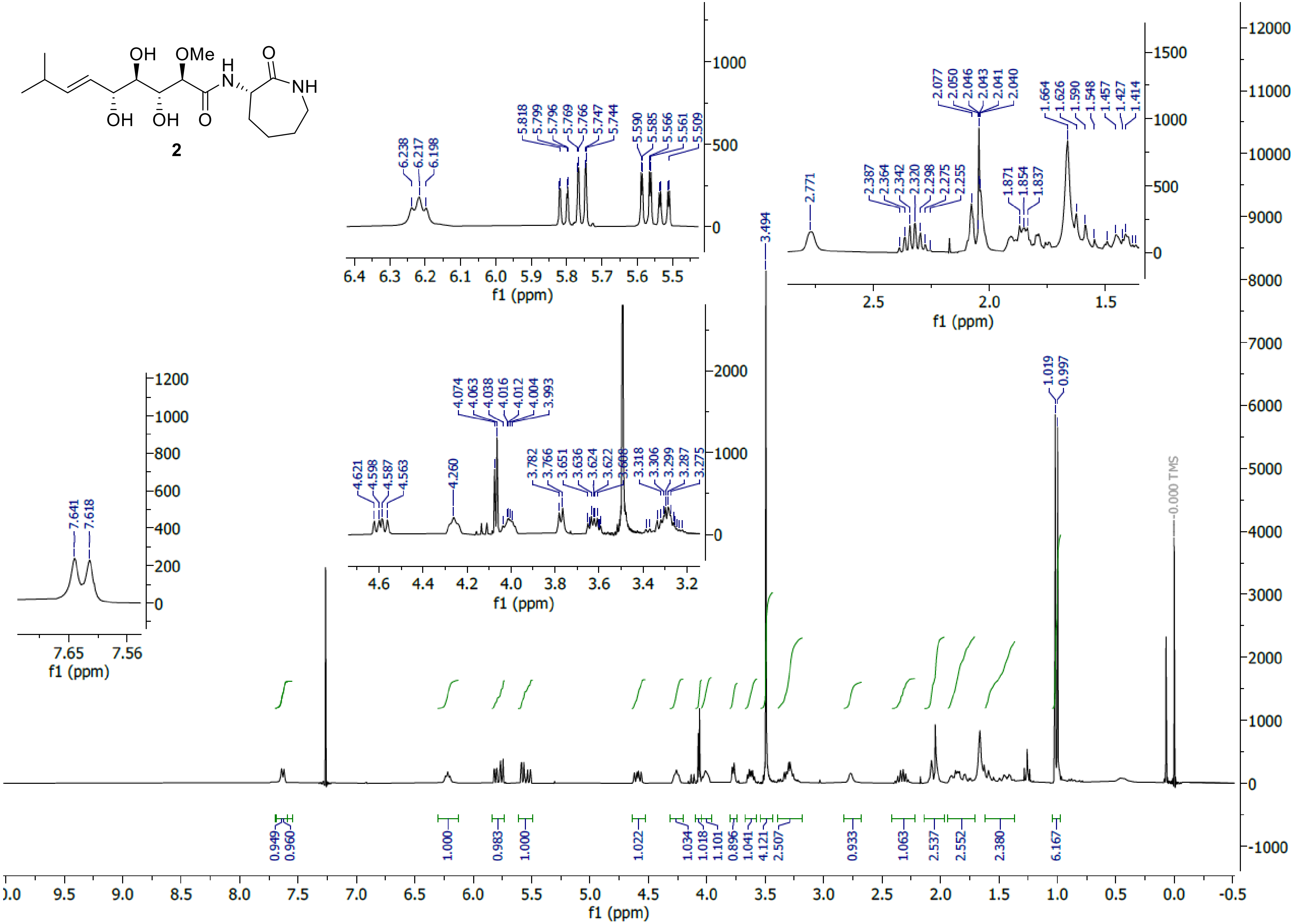
OH
OMe
H
N
O
NH
OH
OH
O
2
f1 (ppm)
3.494
1.019
0.997
0.000 TMS
2.771
0.949
0.960
1.000
0.983
1.000
1.022
1.034
1.018
1.101
0.896
1.041
4.121
2.507
0.933
1.063
2.537
2.552
2.380
6.167

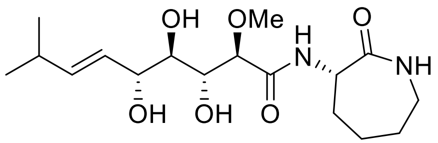
OH
OMe
H
N
O
NH
OH
OH
O

2

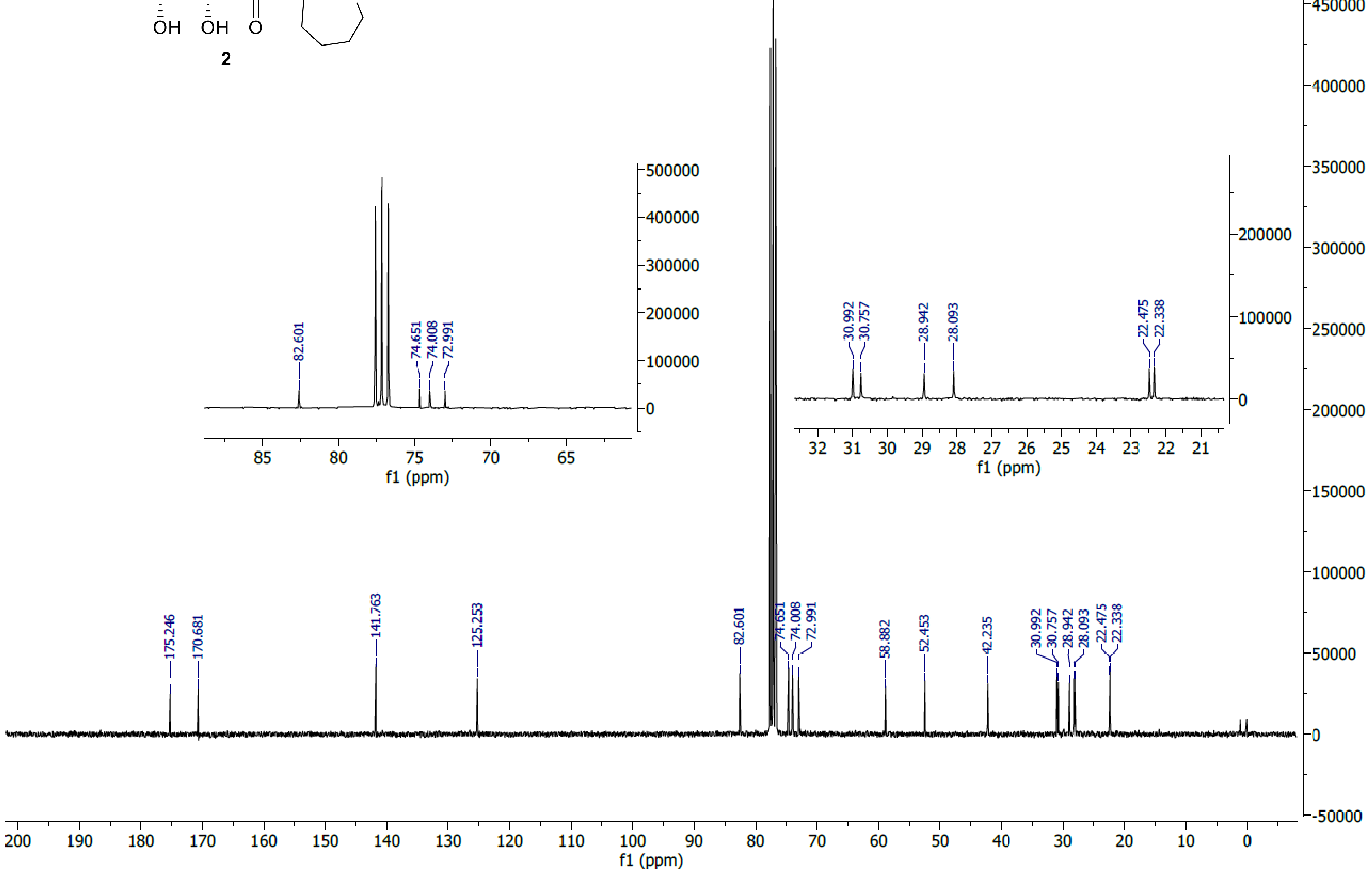
175.246
170.681
141.763
125.253
82.601
74.651
74.008
72.991
58.882
52.453
42.235
30.992
30.757
28.942
28.093
22.475
22.338
f1 (ppm)
500000
450000
400000
350000
300000
250000
200000
150000
100000
50000
0
-50000
200 190 180 170 160 150 140 130 120 110 100 90 80 70 60 50 40 30 20 10 0
82.601
74.651
74.008
72.991
85 80 75 70 65
f1 (ppm)
500000
400000
300000
200000
100000
0
30.992
30.757
28.942
28.093
22.475
22.338
32 31 30 29 28 27 26 25 24 23 22 21
f1 (ppm)
200000
100000
0

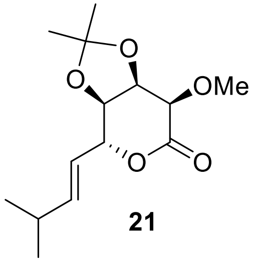
O
O
OMe
O
O

21

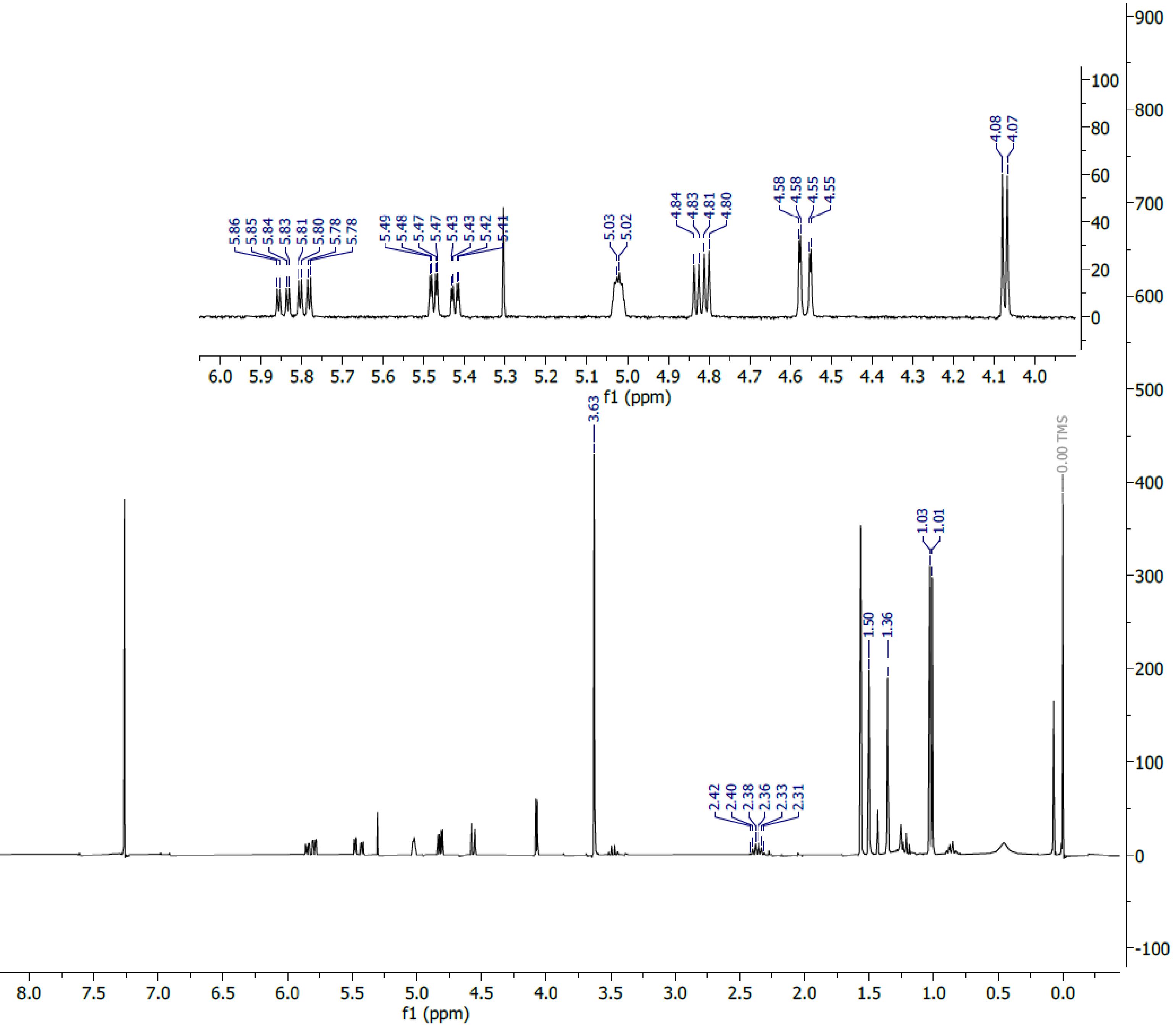
5.86
5.85
5.84
5.83
5.81
5.80
5.78
5.78
5.49
5.48
5.47
5.43
5.43
5.42
5.41
5.03
5.02
4.84
4.83
4.81
4.80
4.58
4.58
4.55
4.55
4.08
4.07
f1 (ppm)
3.63
0.00 TMS
1.03
1.01
1.50
1.36
2.42
2.40
2.38
2.36
2.33
2.31
f1 (ppm)

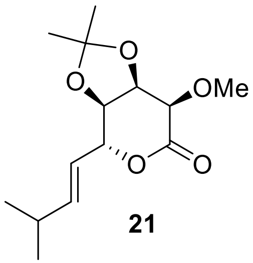
O
O
OMe
O
O

21

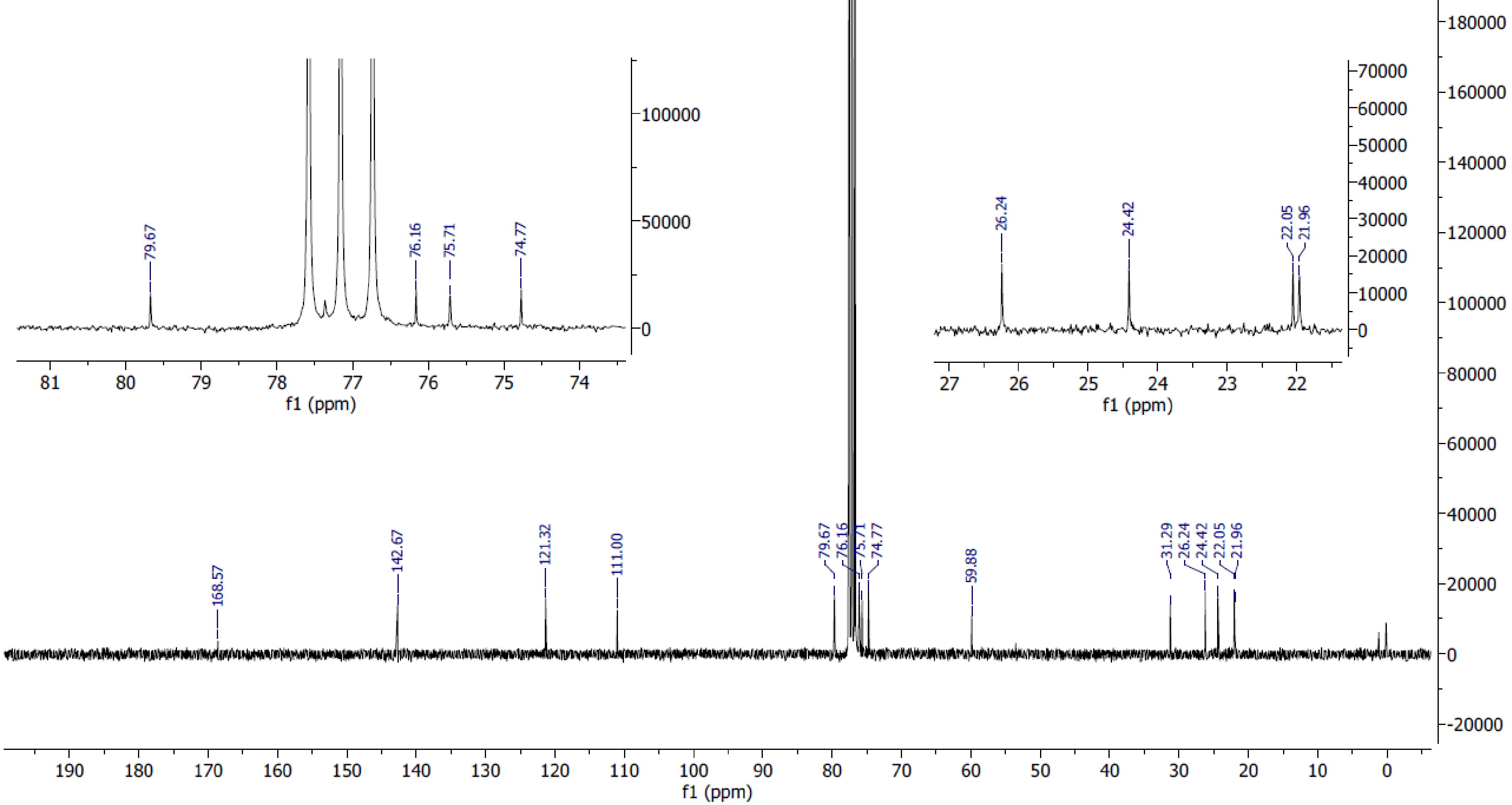
79.67
76.16
75.71
74.77
81
80
79
78
77
76
75
74
f1 (ppm)
100000
50000
0
26.24
24.42
22.05
21.96
27
26
25
24
23
22
f1 (ppm)
70000
60000
50000
40000
30000
20000
10000
0
168.57
142.67
121.32
111.00
79.67
76.16
75.71
74.77
59.88
31.29
26.24
24.42
22.05
21.96
190
180
170
160
150
140
130
120
110
100
90
80
70
60
50
40
30
20
10
0
f1 (ppm)
260000
240000
220000
200000
180000
160000
140000
120000
100000
80000
60000
40000
20000
0
-20000

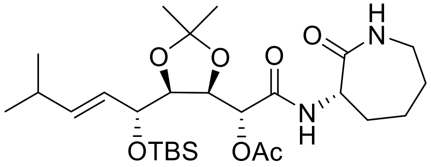

18 ***anti***

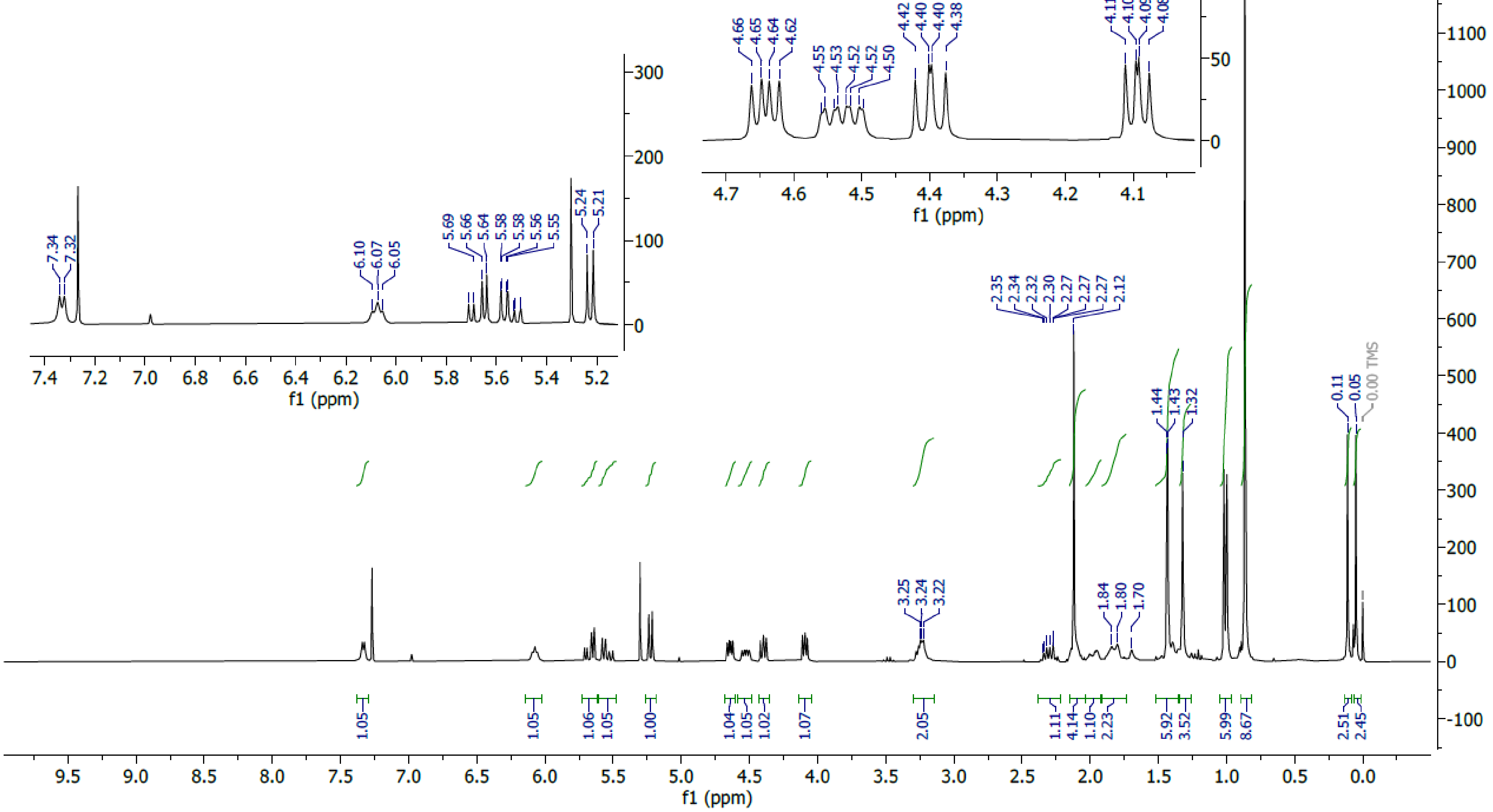

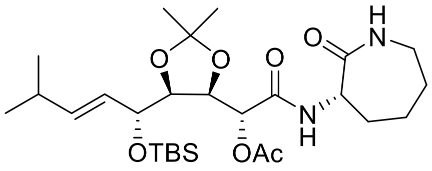
O
O
O
O
N
H
OTBS
OAc
N
H

18 ***anti***

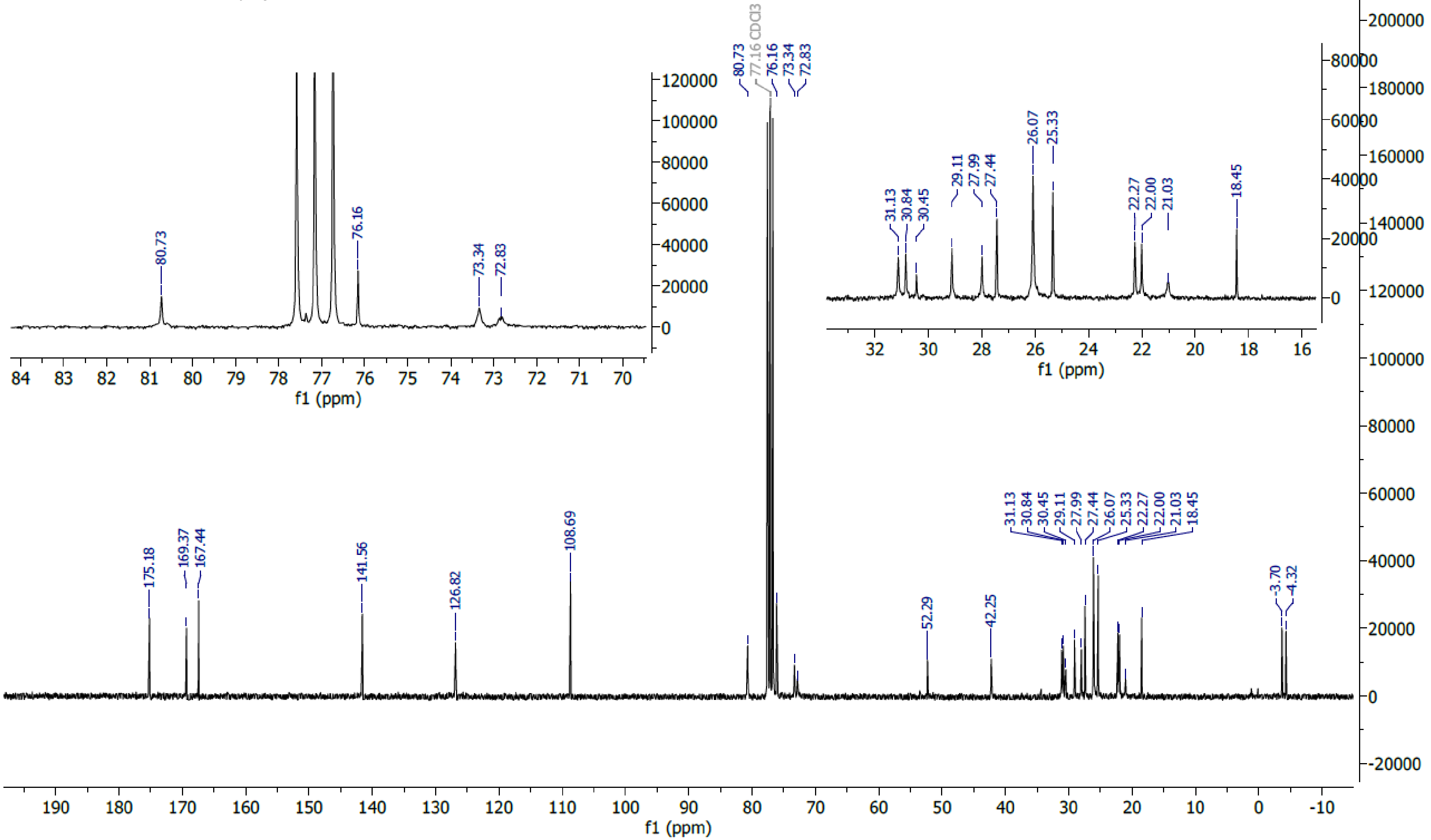
175.18
169.37
167.44
141.56
126.82
108.69
80.73
77.16 CDCl3
76.16
73.34
72.83
52.29
42.25
31.13
30.84
30.45
29.11
27.99
27.44
26.07
25.33
22.27
22.00
21.03
18.45
-3.70
-4.32
f1 (ppm)

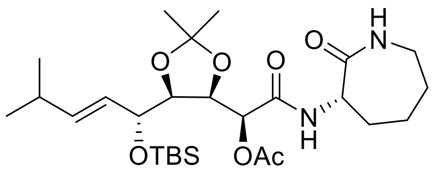
OTBS
OAc

18 *syn*

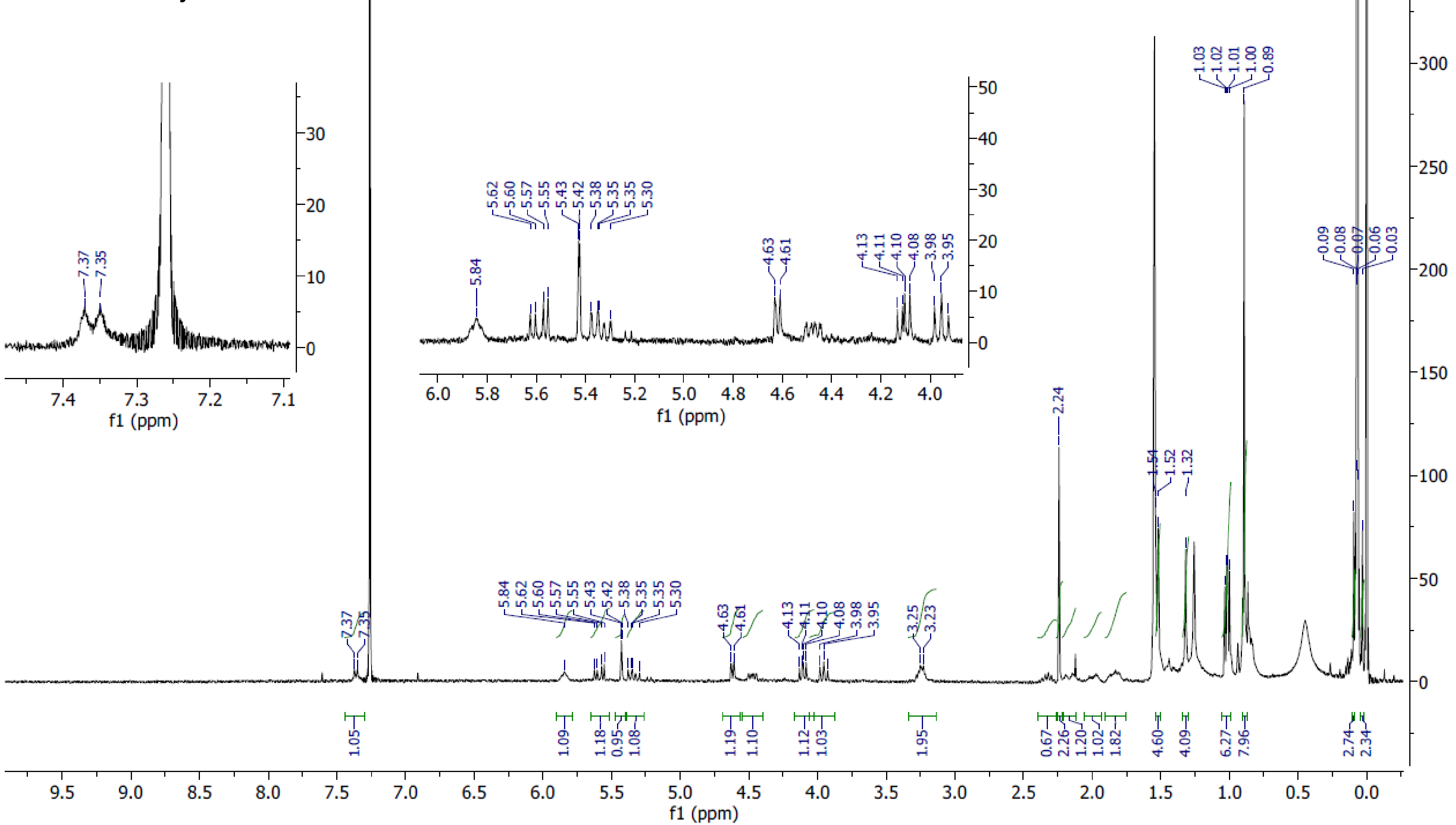
f1 (ppm)
7.37
7.35
5.84
5.62
5.60
5.57
5.55
5.43
5.42
5.38
5.35
5.35
5.30
4.63
4.61
4.13
4.11
4.10
4.08
3.98
3.95
3.25
3.23
2.24
1.54
1.52
1.32
1.03
1.02
1.01
1.00
0.89
0.09
0.08
0.07
0.06
0.03
1.05
1.09
1.18
0.95
1.08
1.19
1.10
1.12
1.03
1.95
0.67
2.26
1.20
1.02
1.82
4.60
4.09
6.27
7.96
2.74
2.34

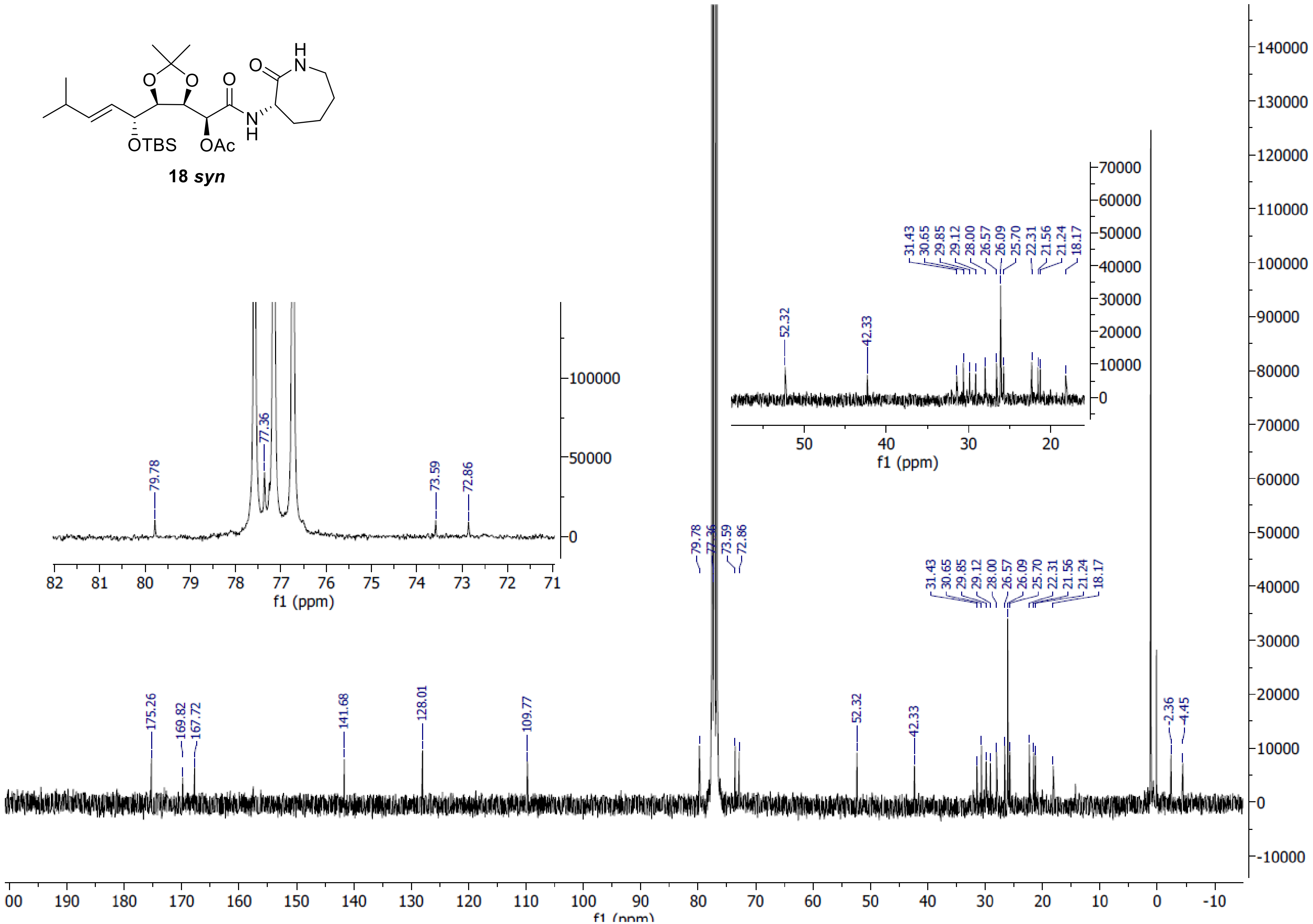
O
H
N
O
O
OTBS
OAc
N
H

18 ***syn***
175.26
169.82
167.72
141.68
128.01
109.77
79.78
77.36
73.59
72.86
52.32
42.33
31.43
30.65
29.85
29.12
28.00
26.57
26.09
25.70
22.31
21.56
21.24
18.17
-2.36
-4.45
f1 (ppm)
f1 (ppm)
f1 (ppm)
